# ZBP1-dependent inflammatory cell death, PANoptosis, and cytokine storm disrupt IFN therapeutic efficacy during coronavirus infection

**DOI:** 10.1126/sciimmunol.abo6294

**Published:** 2022-05-19

**Authors:** Rajendra Karki, SangJoon Lee, Raghvendra Mall, Nagakannan Pandian, Yaqiu Wang, Bhesh Raj Sharma, RK Subbarao Malireddi, Dong Yang, Sanja Trifkovic, Jacob A. Steele, Jon P. Connelly, Gella Vishwanath, Mitnala Sasikala, Duvvur Nageshwar Reddy, Peter Vogel, Shondra M. Pruett-Miller, Richard Webby, Colleen Beth Jonsson, Thirumala-Devi Kanneganti

**Affiliations:** ^1^ Department of Immunology, St. Jude Children's Research Hospital, Memphis, TN 38105, USA; ^2^ UTHSC Regional Biocontainment Laboratory, University of Tennessee Health Science Center, Memphis, TN, USA; ^3^ Department of Infectious Diseases, St. Jude Children's Research Hospital, Memphis, TN 38105, USA; ^4^ Center for Advanced Genome Engineering (CAGE), St. Jude Children’s Research Hospital, Memphis, TN, USA; ^5^ Institute of Pulmonary Medicine and Sleep Disorders, Continental Hospitals, Asian Institute of Gastroenterology, Hyderabad, India; ^6^ Department of Basic Science, Asian Healthcare Foundation, Asian Institute of Gastroenterology, Hyderabad, India; ^7^ Department of Medical Gastroenterology, Asian Institute of Gastroenterology, Hyderabad, India; ^8^ Animal Resources Center and the Veterinary Pathology Core, St. Jude Children's Research Hospital, Memphis, TN 38105, USA; ^9^ Department of Microbiology, Immunology, & Biochemistry, University of Tennessee Health Science Center, Memphis, TN, USA

## Abstract

Severe acute respiratory syndrome coronavirus 2 (SARS-CoV-2), the virus responsible for coronavirus disease 2019 (COVID-19), continues to cause significant morbidity and mortality in the ongoing global pandemic. Understanding the fundamental mechanisms that govern innate immune and inflammatory responses during SARS-CoV-2 infection is critical for developing effective therapeutic strategies. While IFN-based therapies are generally expected to be beneficial during viral infection, clinical trials in COVID-19 have shown limited efficacy and potential detrimental effects of IFN treatment during SARS-CoV-2 infection. However, the underlying mechanisms responsible for this failure remain unknown. In this study, we found that IFN induced ZBP1-mediated inflammatory cell death, PANoptosis, in human and murine macrophages and in the lungs of mice infected with β-coronaviruses, including SARS-CoV-2 and mouse hepatitis virus (MHV). In patients with COVID-19, expression of the innate immune sensor *ZBP1* was increased in immune cells from those who succumbed to the disease compared with those who recovered, further suggesting a link between ZBP1 and pathology. In mice, IFN-β treatment following β-coronavirus infection increased lethality, and genetic deletion of *Zbp1* or its Zα domain suppressed cell death and protected the mice from IFN-mediated lethality during β-coronavirus infection. Overall, our results identify that ZBP1 induced during coronavirus infection limits the efficacy of IFN therapy by driving inflammatory cell death and lethality. Therefore, inhibiting ZBP1 activity may improve the efficacy of IFN therapy, paving the way for the development of new and critically needed therapeutics for COVID-19 as well as other infections and inflammatory conditions where IFN-mediated cell death and pathology occur.

## Teaser

ZBP1 drives pathology during IFN therapy in coronavirus infection.

## INTRODUCTION

Coronavirus disease 2019 (COVID-19), caused by severe acute respiratory syndrome coronavirus 2 (SARS-CoV-2) infection, has led to significant mortality, morbidity and global socioeconomic and psychological distress (*1*, *2*). Severe cases of COVID-19 are characterized by acute respiratory distress syndrome (ARDS), multi-organ failure and death (*3*, *4*). Innate immunity is the first line of host defense against invading viruses, including SARS-CoV-2. Conserved cellular receptors in innate immune cells detect viral DNA and RNA and rapidly induce interferon (IFN) production, which subsequently up-regulates numerous IFN-stimulated genes (ISGs) to limit viral replication (*5*, *6*). However, SARS-CoV-2 can evade antiviral responses by actively interfering with IFN production (*7*–*17*), and the serum of patients with mild and moderate COVID-19 has similar levels of type I and III IFNs compared with healthy patients (*18*). To counteract these evasion mechanisms, IFN treatment has been considered as a therapeutic option for patients with COVID-19. In preclinical studies, treatment with exogenous type I IFN or agonists that induce type I IFN reduce viral load when administered before SARS-CoV-2 infection, but this effect is limited once infection is established (*19*–*22*). These observations suggest that IFN-based therapies have prophylactic, but limited therapeutic, potential. Moreover, the use of IFN-based therapy in the treatment of patients with COVID-19 has yielded mixed results (*23*, *24*). The WHO Solidarity Trial Consortium clinical trial found that type I IFN treatment is not beneficial in treating SARS-CoV-2 infection (*23*). Additionally, a retrospective study found that while administration of IFN-α2b within 5 days of hospitalization is associated with a decrease in mortality, later administration is associated with increased mortality (*24*). Patients with severe COVID-19 are also reported to have increased IFN levels in their serum compared with those with mild infection (*25*). Altogether, these findings indicate that IFN-based therapy may only be beneficial to a certain subset of patients when administered early during the infection and needs to be balanced to avoid pathogenic effects. However, determining the optimal time frame for safe and effective IFN therapy is challenging. Therefore, gaining further insights into the molecular mechanisms of IFN signaling during SARS-CoV-2 infection is essential to inform clinical protocols and improve the efficacy of IFN therapy in COVID-19.

IFN signaling has multiple roles in response to viral infection. In addition to inducing ISGs that have antiviral properties (*26*), IFN signaling also up-regulates ISGs that trigger cell death (*27*, *28*). In COVID-19, there is a positive feedback loop between excessive cell death and IFN signaling that leads to cytokine storm, contributing to multiorgan damage as well as clinical features of disease and mortality (*4*, *29*). Therefore, it is important to understand the connection between IFN, cell death and pathology during SARS-CoV-2 infection to identify ways to maximize the efficacy of IFN-based therapeutic strategies.

One critical ISG that plays a role in cell death during other viral infections is ZBP1, and its complex roles in innate immunity and inflammatory cell death have recently been discovered. During viral infection or IFN-β treatment, ZBP1 is up-regulated in an IFNAR-dependent manner (*28*, *30*). Sensing of viral and endogenous Z-nucleic acids by the ZBP1 Zα domain allows ZBP1 to interact with RIPK3 via RHIM-RHIM homotypic interactions, thereby activating inflammatory cell death (*28*, *30*–*33*). In contrast, up-regulation of ZBP1 by IFN-β treatment alone is not sufficient to induce cell death (*28*). Whether ZBP1 has a role in driving the pathology during severe COVID-19 or whether it has an impact on the efficacy of IFN-based therapies is not known. Here, using CRISPR/CAS9 knockout screening and the analysis of multiple publicly available RNAseq datasets, we identified ZBP1 as a critical cytosolic sensor that drives inflammatory cell death in response to IFN therapy during β-coronavirus infections, including SARS-CoV-2 and mouse hepatitis virus (MHV) infections. IFN-β treatment during SARS-CoV-2 and MHV infections induced robust ZBP1-dependent inflammatory cell death in macrophages. Additionally, single cell transcriptomic analysis showed that *ZBP1* was more abundantly expressed in immune cells from patients who succumbed to COVID-19 than in those who recovered without the need for hospitalization. Furthermore, genetic deletion of *Zbp1* or its Zα domain protected mice from the lethality induced by IFN treatment during β-coronavirus infection. These findings suggest that inhibiting ZBP1 activity could improve the efficacy of IFN therapy during β-coronavirus infection. This improved understanding of the fundamental mechanisms engaged by innate immune receptors in inducing inflammatory responses and cell death during SARS-CoV-2 infection is essential to inform the development of effective therapeutic strategies for the treatment of not only COVID-19, but also other infections and inflammatory diseases.

## RESULTS

### IFN response is delayed during infection with β-coronavirus

**Fig. 1.**
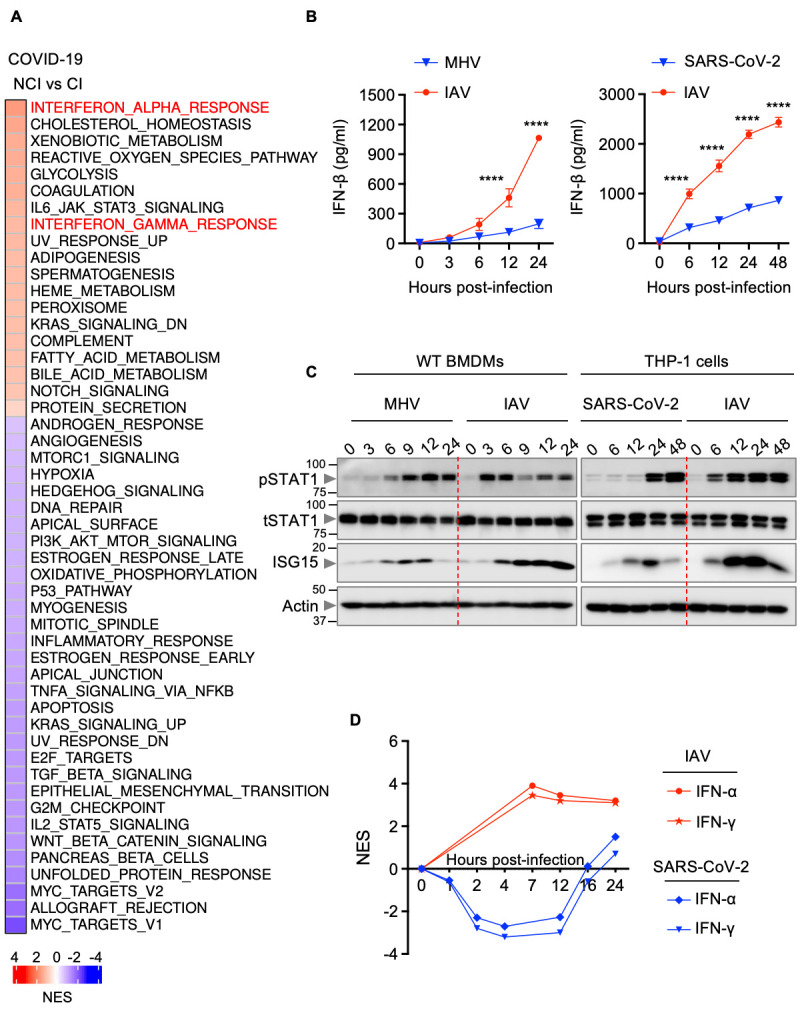
Delayed IFN responses during β-coronavirus infection. (**A**) Heatmap depicting the altered pathways in whole blood cell transcriptomes of critically ill (CI) patients compared with non-critically ill (NCI) patients with COVID-19 (*34*). NES, normalized enrichment scores. (**B**) IFN-β release in the supernatant of wild type (WT) bone marrow-derived macrophages (BMDMs) infected with mouse hepatitis virus (MHV) or influenza A virus (IAV) (left) or THP-1 cells infected with SARS-CoV-2 or IAV (right) for the indicated time. (**C**) Immunoblot analysis of phosphorylated STAT1 (pSTAT1), total STAT1 (tSTAT1) and ISG15 in WT BMDMs infected with MHV or IAV (left) or THP-1 cells infected with SARS-CoV-2 or IAV (right) for the indicated time. Molecular weight marker sizes in kDa are indicated in small font on the left of each blot. (**D**) Kinetics of IFN-α and IFN-γ responses in SARS-CoV-2– or IAV-infected Calu-3 cells (*41*, *104*). Data are representative of at least three independent experiments (**B, C**). ****P < 0.0001. Analysis was performed using the two-way ANOVA (**B**). Data are shown as mean ± SEM.

Understanding the differentially regulated pathways in patients with varying severities of COVID-19 is critical to identify the molecular mechanisms that drive pathogenesis of severe SARS-CoV-2 infection. Using a publicly available dataset, we performed pathway analysis to determine the differentially regulated pathways in non-critically ill (NCI) and critically ill (CI) patients with COVID-19 (*34*). We found that the IFN alpha and IFN gamma response pathways were highly enriched in both NCI and CI patients with COVID-19 compared with healthy patients (**Figure S1A**). Moreover, among the hallmark pathways (*35*, *36*), IFN alpha response was the topmost enriched pathway in CI patients compared with NCI patients with COVID-19 (**Fig. 1A**). We also analyzed the IFN responses to determine differentially regulated ISGs during SARS-CoV-2 infection. Several ISGs were up-regulated in CI patients with COVID-19 compared with healthy patients or NCI patients with COVID-19 (**Figure S1B**). Among the ISGs up-regulated, most of them, including *BATF2, PARP9, DDX60, IFIT3 and IFI44,* have been reported to have antiviral properties (*26*, *37*, *38*), while some are involved in inducing cell death, such as *EIF2AK2* (*27*) *and ZBP1* (*28*, *30*, *32*). However, pathway analysis of whole blood transcriptomes showed that IFN responses in patients with COVID-19 were not as robust as in patients with influenza or sepsis (*39*) (**Figure S2**). Since IFNs can have both beneficial and detrimental effects depending on the context (*40*), we sought to determine the effect of IFNs on cell death during infections with β-coronaviruses and influenza A virus (IAV). Murine bone marrow-derived macrophages (BMDMs) infected with MHV, a prototypical virus of the β-coronavirus genus that mimics many of the key aspects of human β-coronavirus biology, showed delayed dynamics of IFN-β release and STAT1 activation, and reduced ISG up-regulation compared with IAV-infected cells (**Fig. 1B** **and** **1C**). We also found that SARS-CoV-2–infected human THP-1 cells showed delayed IFN responses compared with IAV-infected cells **(** **Fig. 1B** **and** **1C**). Additionally, analysis of a public dataset (*41*) showed early induction of both IFN-α and IFN-γ responses in the lung tissue-derived epithelial cell line Calu-3 upon infection with IAV compared with Calu-3 cells infected with SARS-CoV-2 (**Fig. 1D**). Moreover, patients with COVID-19 are known to exhibit markedly delayed IFN induction (*39*). Altogether, these data suggest that IFN responses are delayed during β-coronavirus infection.

### IFN treatment contributes to cell death and lethality during β-coronavirus infection

**Fig. 2.**
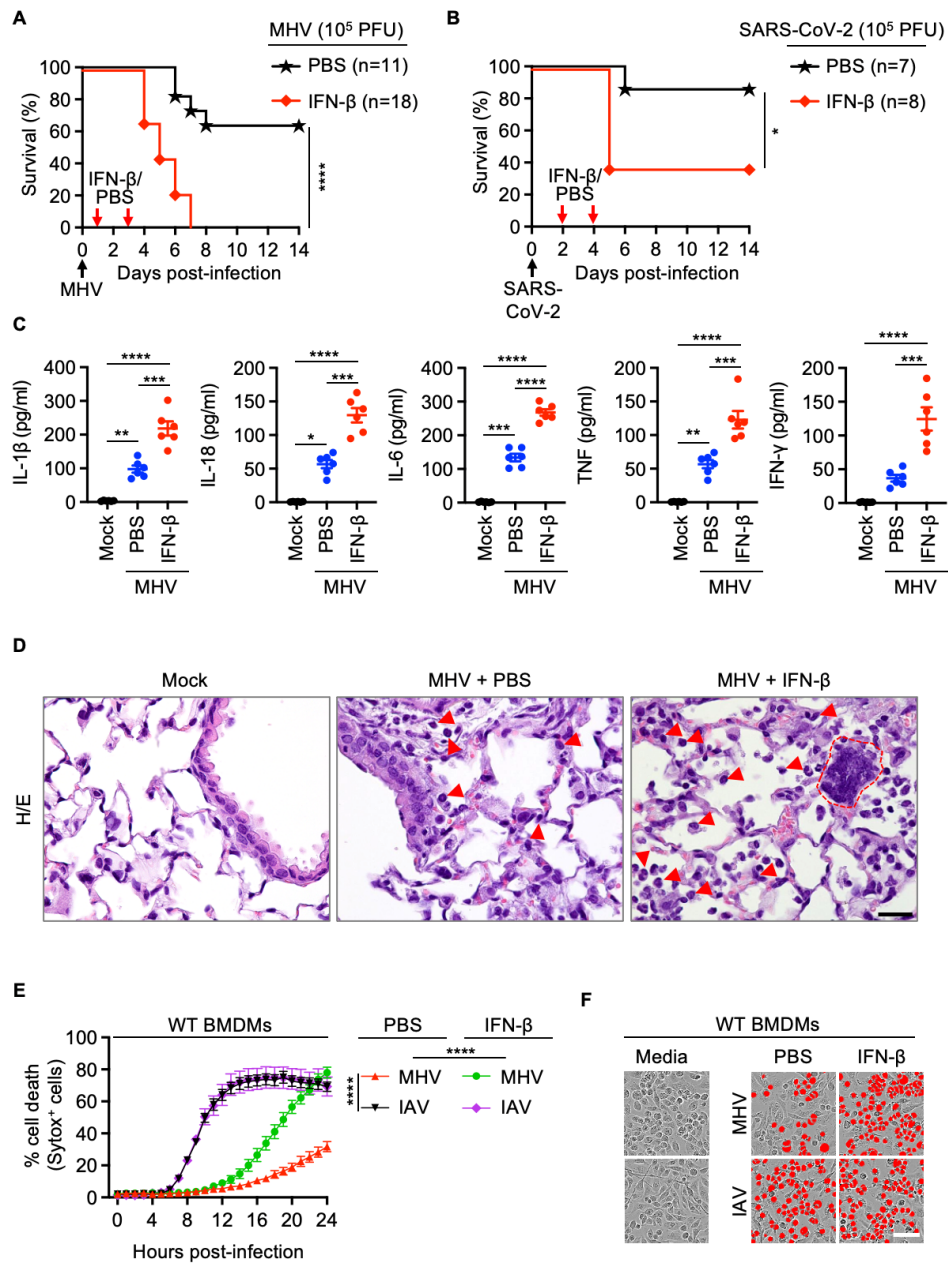
IFN-β treatment drives lethality, cytokine storm and cell death during β-coronavirus infection. (**A**) Survival of 6- to 12-week-old wild type (WT) mice treated with PBS (n = 11) or IFN-β (n = 18) on day 1 and 3 after intranasal infection with mouse hepatitis virus (MHV). (**B**) Survival of 6- to 12-week-old WT mice treated with PBS (n = 7) or IFN-β (n = 8) on day 2 and 4 after intranasal infection with SARS-CoV-2. (**C**) Analysis of IL-1β, IL-18, IL-6, TNF and IFN-γ levels in BALF of uninfected WT mice (mock, n = 6) or PBS- (n = 6) or IFN-β– (n = 6) treated WT mice 3 days after MHV infection. (**D**) Hematoxylin and eosin (H/E) staining of lung samples from WT mice 3 days after MHV infection (PBS- or IFN-β–treated) or no infection (mock). Arrows indicate infiltrating immune cells and dotted red outline represents a syncytial cell. (**E**) Real-time analysis of cell death in PBS- or IFN-β–treated WT bone marrow-derived macrophages (BMDMs) after infection with MHV or influenza A virus (IAV). (**F**) Representative images of cell death in PBS- or IFN-β–treated BMDMs are shown at 24 hours after MHV infection or 16 hours after IAV infection. Scale bar, 50 μm (**D, F**). Data are representative of two (**A, C, D**) or at least three independent experiments (**E, F**). *P < 0.05, **P < 0.01, ***P < 0.001 and ****P < 0.0001. Analysis was performed using the one-way ANOVA (**C**, **E**) or log-rank test (Mantel-Cox) (**A**, **B**). Each symbol represents one mouse (**C**). Data are shown as mean ± SEM. Images are representative of an experiment containing at least 5 biologically independent samples in each group (**D**).

Since IFN responses are reduced in patients with mild and moderate COVID-19 (*18*, *39*), IFN therapy has been suggested as a treatment strategy for patients with COVID-19. However, IFN-based therapy is beneficial only when administered before the onset of clinical symptoms (*24*). On the other hand, delayed IFN-β treatment is pathogenic in a mouse model of MERS-CoV infection (*42*), and administration of IFN-α2b later than 5 days after hospitalization in patients with SARS-CoV-2 is associated with increased mortality (*24*), suggesting that timing of the IFN response relative to virus replication dictates the outcome. To investigate the beneficial or pathological effects of IFN therapy during β-coronavirus infection, we evaluated the effect of IFN treatment during infection in mice. Since infection with β-coronaviruses induced delayed IFN responses (**Figs. 1B** **–** **1D**), and because we wanted to mimic the increased IFN responses observed in CI patients with COVID-19 (**Fig. 1A**), we treated MHV-infected or SARS-CoV-2–infected wild type (WT) mice with recombinant IFN-β and examined the pathology and lethality. MHV-infected mice were dosed with IFN-β on days 1 and 3 post-infection, and SARS-CoV-2–infected mice were dosed on days 2 and 4 post-infection. While 50–60% of WT mice infected with the LD_50_ dose of MHV survived (**Figs. 2A** **and S3A**), all the MHV-infected WT mice receiving IFN-β succumbed to infection within 7 days (**Fig. 2A**). Moreover, 63% of the SARS-CoV-2–infected WT mice receiving IFN-β succumbed to infection within 5 days (**Fig. 2B**), suggesting that IFN-β treatment contributes to lethality during β-coronavirus infection.

Several studies have indicated that IFNs potentiate inflammatory cell death, PANoptosis, leading to cytokine storm and organ damage (*4*, *43*). Consistent with this, at day 3 post-MHV infection, we observed increased production of inflammasome-dependent cytokines IL-1β and IL-18 as well as other pro-inflammatory cytokines in the BALF in WT mice following treatment with IFN-β compared with PBS (**Fig. 2C**). The lungs of IFN-β–treated mice showed widespread septal thickening with scattered syncytial cells and extensive necrosis and loss of bronchiolar epithelium in affected areas during MHV infection (**Figs. 2D** **and S3B, arrows**). The increased production of inflammatory cytokines was accompanied by an increase in alveolar, perivascular and peribronchiolar infiltrates of immune cells, such as macrophages and neutrophils, in the lungs of MHV-infected WT mice treated with IFN-β (**Figs. 2D** **and S3C–S3F**). Additionally, there was augmented cell death, as evidenced by the presence of an increased number of TUNEL^+^ cells, in the lungs of MHV-infected mice treated with IFN-β compared with the infected mice treated with PBS or mock treated mice (**Figure S3E and S3F**), which likely accounts for the extensive necrosis and loss of bronchiolar epithelium observed in these mice. TUNEL positivity, desquamation of bronchial epithelium, marked inflammatory responses and necrosis were also found in lung samples from human patients who succumbed to SARS-CoV-2 infection (**Figure S4**).

To further evaluate the molecular mechanisms of the IFN-induced pathology during infection, we tested whether the increased cell death observed in the lungs of mice treated with IFN-β during MHV infection could be recapitulated in vitro. We found that BMDMs infected with MHV showed delayed cell death compared with cells infected with IAV (**Figs. 2E** **and** **2F**). Moreover, treatment with IFN-β or IFN-γ, but not IL-6, IL-1β or TNF, significantly increased cell death at 24 hours post-MHV infection (**Figure S5**). While addition of IFN-β after infection did accelerate MHV-induced cell death, consistent with our in vivo findings, the supplementation of IFN-β did not change the dynamics of cell death induced by IAV infection (**Figs. 2E** **and** **2F**). Altogether, these data indicate that IFN treatment robustly potentiates the ability of β-coronaviruses to induce cell death, contributing to cytokine storm, lung damage and lethality.

### IFN treatment potentiates inflammatory cell death, PANoptosis, during β-coronavirus infection

**Fig. 3.**
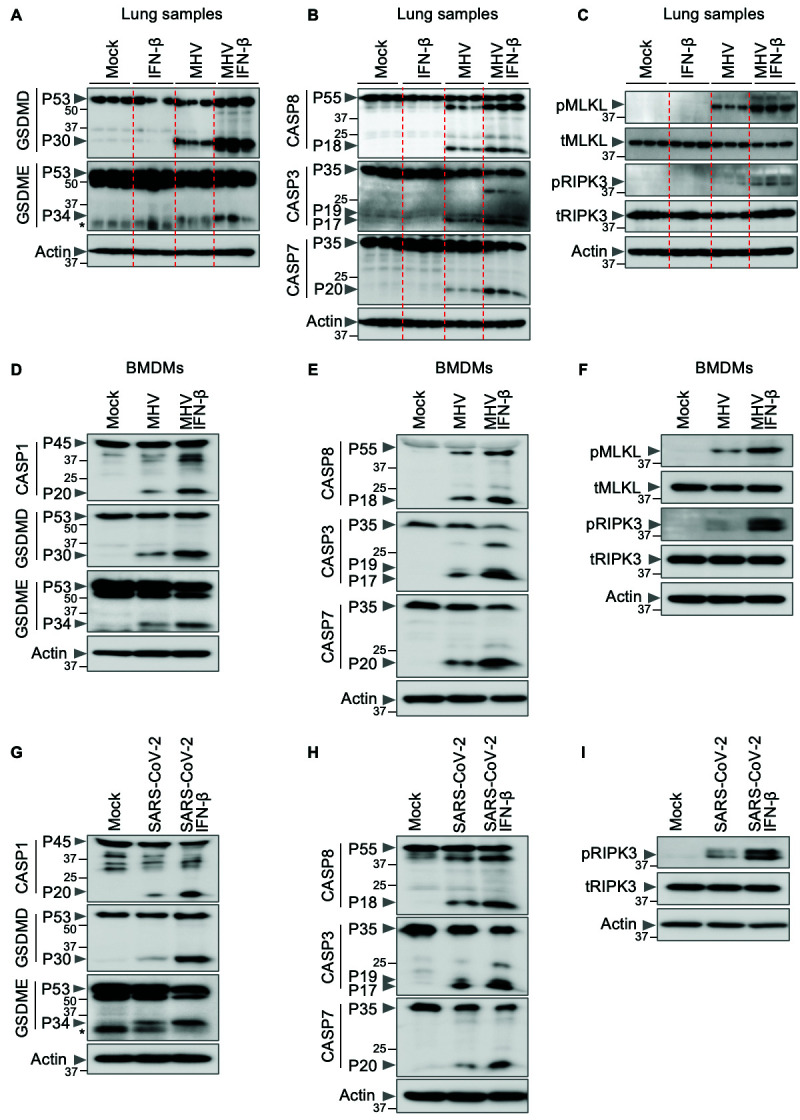
IFN-β promotes inflammatory cell death, PANoptosis, during β-coronavirus infection. (**A**–**C**) Immunoblot analysis of (**A**) pro- (P53) and activated (P30) gasdermin D (GSDMD), pro- (P53) and activated (P34) gasdermin E (GSDME); (**B**) pro- (P55) and cleaved caspase-8 (CASP8; P18), pro- (P35) and cleaved caspase-3 (CASP3; P19 and P17) and pro- (P35) and cleaved caspase-7 (CASP7; P20); and (**C**) phosphorylated MLKL (pMLKL), total MLKL (tMLKL), phosphorylated RIPK3 (pRIPK3) and total RIPK3 (tRIPK3) in the lung samples from mock- or IFN-β–treated wild type (WT) mice with or without mouse hepatitis virus (MHV) infection 3 days post-infection. (**D**–**I**) Immunoblot analysis of (**D**, **G**) pro- (P45) and activated (P20) caspase-1 (CASP1), pro- (P53) and activated (P30) GSDMD, pro- (P53) and activated (P34) GSDME; (**E**, **H**) pro- (P55) and cleaved CASP8 (P18), pro- (P35) and cleaved CASP3 (P19 and P17) and pro- (P35) and cleaved CASP7 (P20); (**F)** pMLKL, tMLKL, pRIPK3 and tRIPK3; and (**I**) pRIPK3 and tRIPK3 in mock- or IFN-β–treated bone marrow-derived macrophages (BMDMs) or THP-1 cells during MHV or SARS-CoV-2 infection, respectively. Actin was used as the internal control. Molecular weight marker sizes in kDa are indicated in small font on the left of each blot. Asterisk denotes non-specific bands (**A, G**). Data are representative of at least three independent experiments.

Cells may undergo multiple forms of cell death, including pyroptosis, apoptosis and necroptosis, depending on the stimulus. Mechanistically, pyroptosis is executed by gasdermin family member-mediated pore formation, which can be induced through inflammasome activation and caspase-1 cleavage of gasdermin D (GSDMD) (*44*, *45*). Apoptosis is induced by the initiator caspases caspase-8/10 or -9, which activate executioners caspase-3 and -7 to drive cell death (*46*, *47*). Necroptosis involves RIPK3-mediated MLKL oligomerization to induce MLKL pores in the membrane and execute cell death (*48*, *49*). Studies have recently identified another cell death pathway called PANoptosis, an inflammatory cell death pathway that integrates components from other cell death pathways. The totality of biological effects in PANoptosis cannot be individually accounted for by pyroptosis, apoptosis or necroptosis alone. PANoptosis is regulated by multifaceted macromolecular complexes termed PANoptosomes (*28*, *30*, *31*, *43*, *50*–*64*). Previous studies have suggested that inflammatory cell death, PANoptosis, can drive cytokine storm during SARS-CoV-2 infection (*4*, *43*). Various pathogens including bacteria, viruses and fungi can trigger cells to undergo PANoptosis (*30*, *51*, *52*, *54*, *59*, *61*, *64*, *65*). Therefore, the increased lethality and lung damage observed in MHV-infected mice upon IFN-β treatment could be due to robust induction of PANoptosis. We therefore biochemically characterized the activation of key PANoptotic proteins (caspase-1, gasdermin D, gasdermin E, caspase-8, -3 and -7, MLKL and RIPK3) in the lungs of infected mice. We found that IFN-β treatment led to increased activation of these molecules in the lungs of WT mice infected with MHV compared with the lungs of infected mice not treated with IFN-β (**Figs. 3A–C**), suggesting that IFN-β treatment potentiates PANoptosis in the lungs of the mice infected with MHV. Similarly, we observed that IFN-β treatment led to increased activation of these molecules in BMDMs infected with MHV (**Figs. 3D–F**) or THP-1 cells infected with SARS-CoV-2 (**Figs. 3G–I**).

Overall, these data indicate that IFN treatment potentiates inflammatory cell death, PANoptosis, that may drive cytokine storm, pathology and lethality in the mice during β-coronavirus infection.

### ZBP1 contributes to pathology and lethality during β-coronavirus infection

**Fig. 4.**
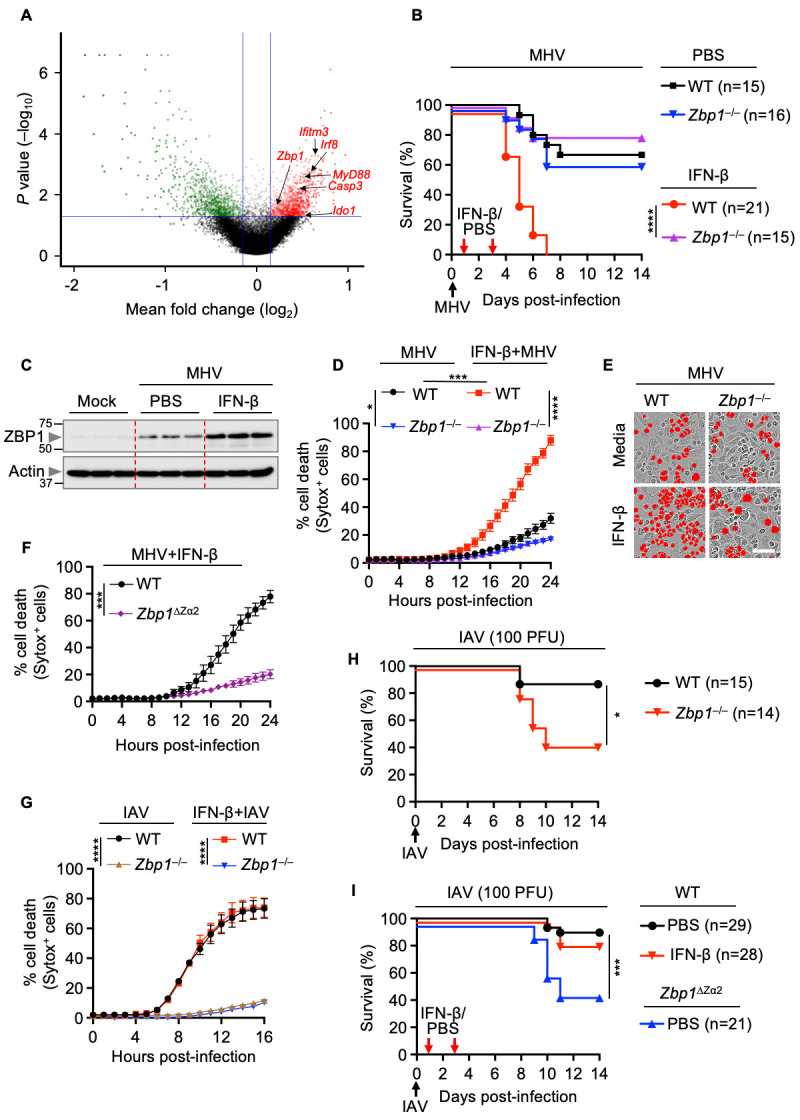
IFN-β–driven lethality, cytokine storm and cell death depend on ZBP1 during β-coronavirus infection. (**A**) Volcano plot showing the genes that are enriched or depleted in immortalized bone marrow-derived macrophages (iBMDMs) following a genome-wide CRISPR/CAS9 knockout screen of cell death induced by mouse hepatitis virus (MHV) infection (MOI 0.2, 24 hours). (**B**) Survival of 6- to 12-week-old wild type (WT) and *Zbp1* ^–/–^ mice treated with PBS (n = 15 for WT and n = 16 for *Zbp1* ^–/–^ mice) or IFN-β (n = 21 for WT and n = 15 for *Zbp1* ^–/–^ mice) on day 1 and 3 after intranasal infection with MHV. (**C**) Immunoblot analysis of ZBP1 in the lung samples from PBS- or IFN-β–treated WT mice 3 days after MHV infection. Molecular weight marker sizes in kDa are indicated in small font on the left of each blot. Actin was used as the internal control. (**D**) Real-time analysis of cell death in MHV-infected WT or *Zbp1* ^–/–^ BMDMs in the presence or absence of IFN-β. (**E**) Representative images of cell death in media- or IFN-β–treated WT or *Zbp1* ^–/–^ BMDMs are shown at 24 hours after MHV infection. Scale bar, 50 μm. (**F**) Real-time analysis of cell death in MHV-infected WT or *Zbp1* ^∆Za2^ BMDMs in the presence of IFN-β. (**G**) Real-time analysis of cell death in influenza A virus (IAV)-infected WT or *Zbp1* ^–/–^ BMDMs in the presence and absence of IFN-β. (**H**) Survival of 6- to 12-week-old WT (n = 15) and *Zbp1* ^–/–^ (n = 14) mice after intranasal infection of IAV. (**I**) Survival of 6- to 12-week-old WT and *Zbp1* ^∆Za2^ mice treated with PBS (n = 29 for WT and n = 21 for *Zbp1* ^∆Za2^ mice) or IFN-β (n = 28 for WT mice) on day 1 and 3 after intranasal infection of IAV. Survival data are pooled from 2 infection experiments (**B, H, I**). All other data are representative of at least three independent experiments. *P < 0.05, ***P < 0.001 and ****P < 0.0001. Analysis was performed using the one-way ANOVA (**D**, **G**), two-tailed *t* test (**F**) or log-rank test (Mantel-Cox) (**B**, **H, I**). Data are shown as mean ± SEM.

Cytosolic sensors that are involved in sensing and inducing inflammatory cell death during β-coronavirus infection remain largely unknown. To identify the innate immune sensors that are required for cell death during β-coronavirus infection, we performed a whole genome CRISPR/CAS9 knockout screen in murine immortalized BMDMs (iBMDMs) using MHV. After generating a pool of cells with individual genes deleted by CRISPR, we infected them with MHV for 24 hours and analyzed the surviving pool of cells to identify the genes that were enriched or depleted in this population. The enriched genes in the surviving pool of cells could be potentially important to play a positive role in inducing cell death during β-coronavirus infection. Since IFN-β treatment contributed to lethality in mice, we then focused our analysis on the ISGs that were significantly up-regulated in the surviving pool (**Fig. 4A** **; Table S1**). Of these ISGs, ZBP1 has been reported to trigger inflammatory cell death in several contexts, including viral infection with IAV (*28*, *30*, *33*, *51*, *64*, *66*). Moreover, *ZBP1* was also identified among the ISGs up-regulated in CI patients with COVID-19 (**Figure S1B**). Therefore, we hypothesized that ZBP1 may be involved in lethality observed in patients with severe COVID-19. To further explore whether ZBP1 expression may correlate with pathology, we re-analyzed publicly available single cell RNAseq datasets to examine the expression of *ZBP1* in immune cells from healthy patients compared with patients with stable COVID-19, who did not require hospitalization, and with those with progressive COVID-19, which led to lethality (*67*). We observed that the expression of *ZBP1* was significantly increased in neutrophils, dendritic cells, macrophages, basophils, NK cells, CD4^+^ T cells and T_reg_ cells from patients with COVID-19 compared with healthy controls (**Figure S6A**). Moreover, patients with progressive COVID-19, who succumbed to infection, had increased *ZBP1* expression in their immune cells, particularly NK cells, CD4^+^ memory and naïve T cells, effector memory CD8^+^ T cells and B memory and naïve cells, compared with patients with stable COVID-19 (**Figure S6B**). Together, these data suggest a positive correlation between *ZBP1* expression and lethality in patients with COVID-19.

Next, we sought to determine the physiological role of ZBP1 in driving lethality and cell death during β-coronavirus infection in the presence or absence of IFN-β treatment. Although IFN-β treatment substantially increased the lethality of MHV infection in WT mice, mice that were deficient in ZBP1 were similarly susceptible to MHV infection-induced lethality regardless of IFN-β treatment (**Fig. 4B**). We found that ZBP1 expression was greatly enhanced in the lungs of MHV-infected mice following IFN-β treatment compared with the lungs of untreated infected mice (**Fig. 4C** **)**. Moreover, we observed decreased production of inflammasome-dependent and -independent pro-inflammatory cytokines in the BALF of MHV-infected *Zbp1* ^–/–^ mice compared with those of WT mice following IFN-β treatment (**Figure S7**). The regulatory role of ZBP1 in vivo was further confirmed by in vitro findings that the robust cell death observed in MHV-infected BMDMs in response to IFN treatment was reduced in ZBP1-deficient cells (**Figs. 4D** **and** **4E**). Sensing of viral or endogenous Z-RNA by the Zα domains of ZBP1 triggers NLRP3 inflammasome activation, inflammatory cell death and perinatal lethality in mice, indicating that the Zα domains are crucial to regulate the immune responses driven by ZBP1 (*31*–*33*). Therefore, we investigated the role of the Zα2 domain of ZBP1 in driving the cell death induced by MHV with IFN-β treatment. Similar to *Zbp1* ^–/–^ BMDMs, MHV-infected cells lacking the Zα2 domain of ZBP1 showed reduced cell death compared with that of WT BMDMs after treatment with IFN-β (**Fig. 4F** **)**. However, IFN-β treatment did not affect the ZBP1-dependent cell death in BMDMs during IAV infection (**Fig. 4G**), likely because IAV infection naturally induces more rapid IFN production than β-coronavirus infection, making the additional IFN ineffective. Although *Zbp1* ^–/–^ and *Zbp1* ^ΔZα2^ mice showed increased susceptibility to IAV infection with respect to WT mice, IFN-β treatment did not change the outcome in WT mice during IAV infection (**Figs. 4H** **and** **4I**).

Altogether, our results show that ZBP1 drives cell death, cytokine storm and lethality in β-coronavirus-infected mice in response to IFN-β treatment.

### ZBP1 triggers inflammatory cell death, PANoptosis, and cytokine storm during β-coronavirus infection

**Fig. 5.**
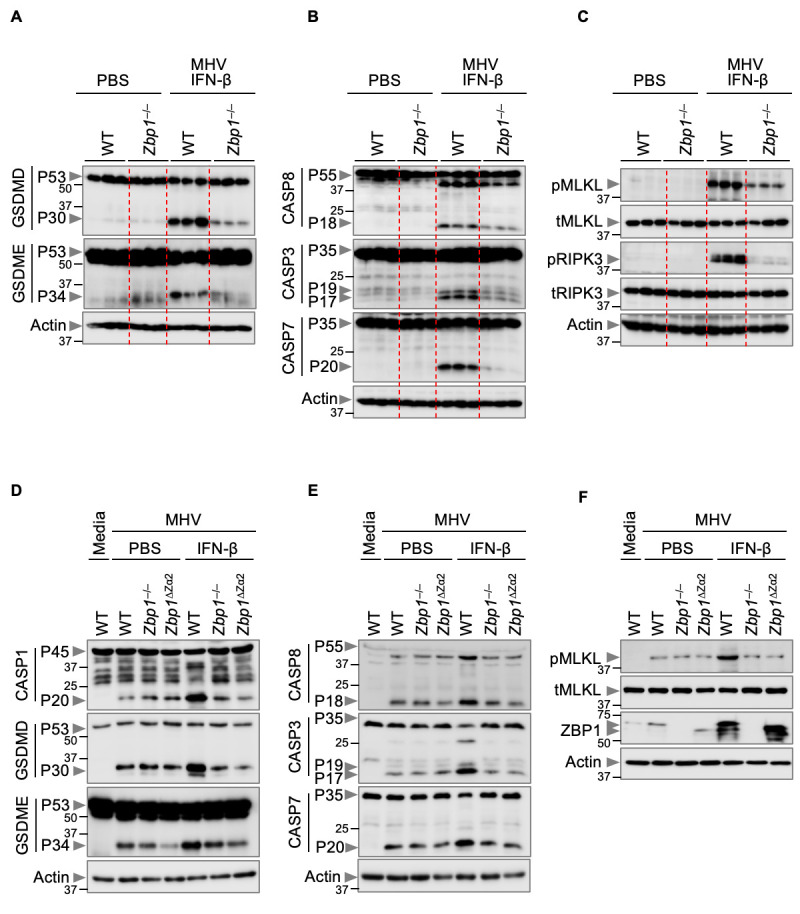
Zα domain of ZBP1 drives PANoptosis mediated by IFN-β during β-coronavirus infection. (**A**–**C**) Immunoblot analysis of (**A**) pro- (P53) and activated (P30) gasdermin D (GSDMD), pro- (P53) and activated (P34) gasdermin E (GSDME); (**B**) pro- (P55) and cleaved caspase-8 (CASP8; P18), pro- (P35) and cleaved caspase-3 (CASP3; P19 and P17) and pro- (P35) and cleaved caspase-7 (CASP7; P20); and (**C**) phosphorylated MLKL (pMLKL), total MLKL (tMLKL), phosphorylated RIPK3 (pRIPK3) and total RIPK3 (tRIPK3) in the lung samples from PBS-treated mice or mouse hepatitis virus (MHV)-infected wild type (WT) and *Zbp1* ^–/–^ mice treated with IFN-β harvested 3 days after infection. (**D**–**F**) Immunoblot analysis of (**D**) pro- (P45) and activated (P20) caspase-1 (CASP1), pro- (P53) and activated (P30) GSDMD, pro- (P53) and activated (P34) GSDME; (**E**) pro- (P55) and cleaved (P18) CASP8, pro- (P35) and cleaved (P19 and P17) CASP3 and pro- (P35) and cleaved (P20) CASP7; and (**F**) pMLKL, tMLKL and ZBP1 in PBS- or IFN-β–treated WT, *Zbp1* ^–/–^ and *Zbp1* ^∆Za2^ bone marrow-derived macrophages (BMDMs) during MHV infection. Actin was used as the internal control. Data are representative of at least three independent experiments.

ZBP1 has been shown to trigger cell death by activating PANoptosis in cells during infection with viruses and fungi (*30*, *31*, *51*, *61*, *64*). Combined with our observation that PANoptosis was occurring in the lungs of MHV-infected mice in response to IFN-β treatment (**Figs. 3A–C**), this suggests that ZBP1-mediated PANoptosis may contribute to the lung pathology, cytokine storm and lethality in MHV-infected mice during IFN-β treatment. In support of this hypothesis, we found that the robust activation of PANoptotic markers was reduced in the lungs of MHV-infected *Zbp1* ^–/–^ mice compared with those of WT mice during IFN-β treatment (**Figs. 5A** **–** **5C**). Additionally, consistent with the reduced cell death observed in *Zbp1* ^–/–^ and *Zbp1* ^ΔZα2^ BMDMs (**Figs. 4D** **–** **4F**), these cells also showed impaired activation of PANoptotic effectors in response to IFN-β treatment (**Figs. 5D** **–** **5F**). Similarly, *ZBP1*-silenced THP-1 cells infected with SARS-CoV-2 showed reduced activation of PANoptotic effectors when treated with IFN-β compared with control infected and IFN-β–treated cells (**Figure S8A–S8C**). Together, these results indicate that ZBP1, and specifically its Zα2 domain, is required for β-coronavirus–induced inflammatory cell death in response to IFN-β treatment.

ZBP1 activation leads to its interaction with RIPK3 and recruitment of caspase-8 and caspase-6 to form a cell death signaling scaffold, termed the ZBP1-PANoptosome, that drives NLRP3 inflammasome activation and cell death (*30*, *31*, *61*, *68*). We therefore assessed whether RIPK3 and caspase-8 had any role in the β-coronavirus–induced cell death pathway during IFN-β treatment. Similar to *Zbp1* ^–/–^ or *Zbp1* ^ΔZα2^ BMDMs, cells lacking RIPK3 showed reduced cell death and activation of PANoptotic molecules compared with those of WT BMDMs during MHV infection in response to IFN-β treatment (**Figure S8D–S8H**). We did not observe significant differences in the extent of cell death and activation of PANoptosis between *Ripk3* ^–/–^ and *Ripk3* ^–/–^ *Casp8* ^–/–^ BMDMs (**Figure S8D–S8H**), indicating that the phenotype observed in *Ripk3* ^–/–^ *Casp8* ^–/–^ BMDMs is largely attributed to the RIPK3 deficiency, as caspase-8 is upstream of RIPK3. Together, these findings suggest that ZBP1-mediated activation of RIPK3-dependent PANoptosis is responsible for the cell death induced by IFN-β treatment during β-coronavirus infection.

## DISCUSSION

Vaccines remain the best approach to end the COVID-19 pandemic (*69*). However, the efficacy of the vaccines is challenged by the emergence of new SARS-CoV-2 variants (*70*). Use of drugs including the antivirals remdesivir, the nirmatrelvir and ritonavir combination and molnupiravir, as well as anti-inflammatory or immunomodulatory agents, such as dexamethasone, tocilizumab and baricitinib, have been shown to improve outcomes in patients with COVID-19 (*71*–*75*). However, these agents typically provide limited benefits to a subset of patients, and global availability is inadequate; the morbidity and mortality caused by COVID-19 remain high (*1*). Given the potency of IFNs in controlling viral replication, several clinical trials using IFNs alone or in combination with other antiviral drugs have been initiated to treat patients infected with SARS-CoV-2. Coronaviruses, including SARS-CoV-2 and MHV, induce a delayed IFN response (*19*), because of which the viruses likely undergo active replication to establish infection. However, preclinical data have shown that this delayed response can be overcome by treatment with exogenous IFNs or IFN-inducing agonists (*19*). The observed benefits of early type I IFN responses induced by some agonists in preclinical models suggest that IFN-based therapies may be effective for the prevention and treatment of COVID-19 (*19*–*22*, *76*, *77*). However, the use of IFN therapy, which acts by decreasing viral replication, has shown mixed responses in clinical trials (*23*, *24*, *78*–*83*). A recent double blinded and placebo-controlled clinical trial (ACTT-3) showed no significant difference in patient outcomes with the combination of IFN-β-1a plus remdesivir compared with remdesivir alone, suggesting no clinical benefits of IFN therapy in patients with COVID-19 (*23*). Moreover, patients who required supplemental oxygen or non-invasive ventilation tended to develop adverse outcomes, particularly worsening of respiratory function, in response to IFN-β-1a administration in this trial (*23*). It is possible that IFN treatment may increase the inflammatory response, leading to more severe respiratory disease in these patients.

In vivo, the lungs of MHV-infected mice had increased immune cell infiltration, inflammatory cytokine levels and PANoptosis in response to IFN treatment compared with PBS treatment, and the IFN-treated mice were more likely to succumb to infection. Similar survival was observed in SARS-CoV-2–infected mice, where IFN treatment significantly increased mortality. In vitro, MHV-infected BMDMs or SARS-CoV-2–infected monocytes showed increased PANoptosis following IFN treatment compared with mock treatment. BMDMs or THP-1 cells were treated with IFN-β after 5 hours of MHV or SARS-CoV-2 infection. However, the effect of IFN-β treatment at different time points after β-coronavirus infection has not been investigated in this study, and the outcomes may change depending on when IFN-β treatment is given. Moreover, the dose of IFN-β is another factor that may influence the outcome. In addition, since epithelial cells are the primary cells that are infected by β-coronaviruses, future work to determine the activation of PANoptosis in cell types other than myeloid cells, such as epithelial and endothelial cells, would provide additional information about the cell types that respond to IFNs and are responsible for driving detrimental effects during β-coronavirus infections. Additionally, PANoptosis in macrophages activates both GSDMD and GSDME pore-forming molecules during SARS-CoV-2 infection, but it is possible these molecules may have PANoptosis-independent functions in certain cell types. For instance, high expression of GSDMD has been associated with neutrophil extracellular trap (NET) structures in the lungs of patients with COVID-19. Pharmacological inhibition of GSDMD through treatment with the inhibitor disulfiram reduces NET release and organ damage in a mouse model of SARS-CoV-2 infection, suggesting that GSDMD-dependent NETosis plays a critical role in COVID-19 immunopathology (*84*). These functions require further study.

IFNs synergize with TNF to induce inflammatory cell death, leading to organ and tissue damage in cytokine storm-associated mouse models (*4*, *43*). Moreover, IFNs can damage the lung epithelial barrier and impair lung epithelial regeneration during viral infection (*85*). In addition, IFN treatment induces sustained expression of ISGs (*19*), which include both anti-viral as well as inflammatory cell death-driving molecules, such as ZBP1. Not all patients infected with SARS-CoV-2 showed increased IFN responses and ZBP1 expression. However, we observed that critically ill patients with COVID-19 showed increased IFN responses and ZBP1 expression compared to non-critical patients with COVID-19, suggesting that IFNs and ZBP1 are associated with the disease progression and mortality. Indeed, the loss of ZBP1 substantially protected the β-coronavirus–infected mice from lung damage, cytokine storm and lethality during IFN treatment, highlighting the detrimental effect of ZBP1 in the disease progression. The use of IFN therapy in patients infected with SARS-CoV-2 would therefore mimic the increased and sustained ZBP1 expression observed in the critically ill patients with COVID-19, which could result in multi-organ damage, cytokine storm and mortality in patients. In contrast to its role in response to IFN treatment during β-coronavirus infection, we observed that ZBP1 was critical to the host defense against IAV infection irrespective of IFN treatment. This is likely due to the ability of IAV to induce rapid IFN responses and ZBP1 expression, which facilitates viral clearance via molecular mechanisms that include ZBP1-dependent inflammatory cell death (*30*, *86*, *87*). However, in addition to clearing the virus, dysregulated inflammatory cell death leads to cytokine storm and organ damage (*4*, *43*). Notably, MHV-infected mice succumbed to infection upon IFN treatment despite the fact that IFN should inhibit viral replication, suggesting that inflammatory cell death, rather than the virus, is responsible for driving pathological effects. However, these consequential pathological effects of IFN therapy during β-coronavirus infection can be blocked by inhibiting ZBP1 activity. Therefore, inhibiting ZBP1 activity may improve the beneficial effects of IFN-β therapy during β-coronavirus infection. Moreover, the associated side effects and inconvenience associated with exogenous IFN administration can be overcome by administering agonists that engage DNA sensing pathways such as cGAS and STING or RNA sensing pathways such as MDA5, RIG-I and MAVS to produce IFNs endogenously (*19*–*22*). The sustained and late IFN production induced by the cGAS-STING pathway also contributes to collateral host responses and tissue damage leading to lethality in SARS-CoV-2–infected mice (*88*), which could be due to ZBP1-dependent inflammatory cell death, and these factors would need to be considered when designing therapeutic strategies.

Overall, our results suggest that IFN-based treatments for COVID-19 face therapeutic challenges due to ZBP1-mediated inflammatory cell death that contributes to cytokine storm, tissue damage and ultimately lethality. Combination strategies that block ZBP1 while also providing IFN treatment may therefore be beneficial for patient outcomes and should be pursued for further research and development. This improved understanding of the mechanistic basis of innate immune sensing, IFN-mediated pathology and inflammatory cell death during SARS-CoV-2 infection informs COVID-19 therapeutic development and provides fundamental molecular details that should be evaluated across interferonopathies.

## MATERIALS AND METHODS

### Study design

The aim of this study was to investigate the molecular mechanism underlying the failure of IFN therapy during SARS-CoV-2 infection. We used MHV, a prototypical virus of the β-coronavirus genus that mimics many of the key aspects of human β-coronavirus biology, as well as SARS-CoV-2 to infect WT mice in the presence and absence of IFN-β and determined pathology and lethality. Furthermore, we investigated the effect of IFN treatment on mouse BMDMs infected with MHV and human THP-1 cells infected with SARS-CoV-2 in inducing inflammatory cell death, PANoptosis. We also performed a CRISPR screen to identify key molecules involved in cell death in response to MHV infection. Sample sizes used in each experiment are detailed in the figure legends.

### Mice

C57BL/6J [wild type (WT)], *Zbp1* ^−/−^ (*89*), *Zbp1* ^∆Zα2/∆Zα2^ (*31*), *Ripk3* ^–/–^ (*90*) and *Ripk3* ^–/–^ *Casp8* ^–/–^ (*91*) mice have been described previously. All mice were bred and maintained in a specific pathogen-free facility at the Animal Resources Center at St. Jude Children’s Research Hospital and were backcrossed to the C57BL/6 background (J substrain) for at least 10 generations. Both male and female mice were used in this study; age- and sex-matched 6- to 12-week-old mice were used for in vivo and in vitro studies and were randomly assigned to different treatment groups/stimulations. Cohoused animals were used for in vivo MHV infections. Mice were maintained with a 12 hours light/dark cycle and were fed standard chow. Animal studies were conducted under protocols approved by the St. Jude Children’s Research Hospital committee on the Use and Care of Animals or with the approval of the Institutional Animal Care and Use Committee of University of Tennessee Health Science Center (UTHSC IACUC Protocol #20-0132).

### Cell culture

Primary bone marrow-derived macrophages (BMDMs) from mice were cultivated for 6 days in IMDM (Thermo Fisher Scientific, 12440061) supplemented with 10% FBS (Biowest, S1620), 30% L929-conditioned media, 1% non-essential amino acids (Thermo Fisher Scientific, 11140-050) and 1% penicillin and streptomycin (Thermo Fisher Scientific, 15070-063). BMDMs were then seeded into 12-well plates at a density of 1 million cells per well and incubated overnight before use. THP-1 cells were grown in RPMI 1640 with 10% FBS and differentiated into macrophages in RPMI 1640 medium containing 20% FBS and 100 ng/ml PMA for 2 days.

### Virus culture

The MHV (A59 strain from Dr. Channappanavar) (*92*) was amplified in 17Cl-1 cells as previously described (*93*). Briefly, 17Cl-1 cells were inoculated with MHV-A59 at a MOI of 0.1. At 48 hours post-infection, the whole flask with media and cells was frozen at –80°C and then thawed at 37°C. After repeating the freezing–thawing cycle twice, the supernatant was collected and centrifuged at 2,000 × g for 10 min to remove the cell debris. Then the virus was purified by ultracentrifugation at 30,000 rpm for 1 hour, after which the pellets were resuspended with fresh medium. The virus titer was measured by plaque assay in 17Cl-1 cells. SARS-CoV-2 for in vitro analyses (strain USA-WA1/2020 isolate; BEI Resources cat# NR-52281) was obtained from BEI Resources (https://www.beiresources.org) and amplified in Vero E6 cells. SARS-CoV-2 for in vivo analyses (isolate hCoV-19/USA/MD-HP01542/2021; Lineage B.1.351, NR-55282) was obtained from BEI Resources. SARS-CoV-2 B.1.351 (Beta) seed stocks were amplified in VeroE6/TMPRSS2 cells in infection media comprising of MEM with Earle’s salts and L-glutamine with 2% FBS and 1% penicillin-streptomycin. Amplified stock virus was stored at –80°C until used. The influenza A virus (A/Puerto Rico/8/34, H1N1 [PR8]) was prepared as previously described (*61*) and propagated from 11-day-old embryonated chicken eggs by allantoic inoculation. Influenza A virus titer was measured by plaque assay in MDCK cells.

### Cell stimulation and infection

For MHV (16 hours or 24 hours; MOI 0.5, unless otherwise noted) and IAV (16 hours; MOI 5, unless otherwise noted) infections, cells were infected in DMEM plain media without glutamic acid and sodium pyruvate (Sigma, D6171). For SARS-CoV-2 infection, THP-1 cells were seeded in 6-well plates and activated with 10 ng/ml of PMA for 2–3 days. THP-1 cells were infected by incubating with SARS-CoV-2 (MOI 0.1 for 1 hour) in a CO_2_ incubator at 37°C. After, cells were washed once with PBS and replenished with media. Infected cells were then cultured in a CO_2_ incubator at 37°C. For cytokine treatment, BMDMs were stimulated with the following concentrations of cytokines: 20 ng/mL of IL-6 (Peprotech, 212-16), 20 ng/mL of IL-1β (R&D, 201-LB-025), 25 ng/mL of TNF (Peprotech, 315-01A), 50 ng/mL of IFN-γ (Peprotech, 315-05) or 50 ng/mL of IFN-β (PBL Assay, 12400-1) for the indicated time. Human THP-1 macrophages were stimulated with 50 ng/mL of IFN-β (PBL Assay, 11410-2) for the indicated time. Cytokines were added to BMDMs or THP-1 cells 5 hours after infections.

### siRNA-mediated gene silencing

The Accell human siRNAs against *ZBP1* (E-014650-00-0010) were purchased (Horizon). THP-1 macrophages were transfected with siRNA using Accell siRNA delivery media according to the manufacturer’s instructions (Horizon). As a negative control, non-targeting control siRNA (D-001910-01-50) was used.

### In vivo infection

Age- and sex-matched, 6- to 12-week-old WT, *Zbp1* ^–/–^ and *Zbp1* ^∆Zα2/∆Zα2^ mice were used for infections. Mice were anesthetized with 250 mg/kg Avertin and then infected intranasally. For determination of the LD_50_ dose of MHV, mice were infected with 50 μl PBS containing ~10^4^, ~10^5^ or ~10^6^ PFU of MHV. For subsequent infections, mice were infected with 50 μl PBS containing ~10^5^ PFU of MHV or ~100 PFU of IAV. The MHV-infected mice were injected intraperitoneally with 200 μl of PBS or IFN-β (1 μg/mouse) diluted in PBS on day 1 and day 3 post-infection. The mice were monitored over a period of 14 days for survival. For collection of BALF or lungs, mice were dosed with PBS or IFN-β as described above on day 1, and samples were collected at day 3 post-infection.

SARS-CoV-2 infections were conducted in accordance with the approval of the Institutional Animal Care and Use Committee of University of Tennessee Health Science Center (UTHSC IACUC Protocol #20-0132). The SARS-CoV-2 related experiments were performed in Animal Biosafety Level 3 conditions within the UTHSC Regional Biocontainment Laboratory according to UTHSC RBL Standard Operating Procedures and safety manuals. Each mouse was intranasally infected with SARS-CoV-2 B.1.351 variant at 1 × 10^5^ PFU in a total volume of 100 μl (50 μL/nare) at day 0. The SARS-CoV-2–infected mice were injected intraperitoneally with 150 μl of PBS or IFN-β diluted in PBS on day 2 and day 4 post-infection. Mice were weighed and monitored daily in the morning. The mice health status was evaluated once per day using the clinical scoring system approved by UTHSC IACUC, twice daily upon clinical manifestation. The mice were monitored over a period of 14 days for survival.

### Histopathology

For murine samples, lungs were fixed in 10% formalin, then processed and embedded in paraffin by standard procedures. Sections (5 μM) were stained with hematoxylin and eosin (H/E) and examined by a pathologist blinded to the experimental groups. For immunohistochemistry, formalin-fixed paraffin-embedded lungs were cut into 4 μM sections. F4/80 (Cell Signaling, D2SR9) and Ly-6B.2 (Novus, NBP2-13077) staining was performed according to the manufacturer’s instructions. TUNEL (terminal deoxynucleotidyl transferase deoxyuridine triphosphate nick-end labeling) staining was performed using the Dead-End kit (Promega, PRG7130) according to the manufacturer’s instructions.

For human lung samples, de-identified autopsy lung samples were provided by the Asian Institute of Gastroenterology as paraffin embedded tissue blocks. Sectioning (4 μm) and immunohistochemistry were performed by HistoWiz with hematoxylin and eosin, the rabbit polyclonal anti–SARS-CoV-2 Nucleocapsid (N) protein antibody (GeneTex; GTX635686), and TUNEL (Promega) staining following standard protocols.

### Immunoblot analysis

Immunoblotting was performed as described previously (*94*). Briefly, for caspase analysis, BMDMs were lysed along with the supernatant using 50 μl caspase lysis buffer (1 × protease inhibitors, 1 × phosphatase inhibitors, 10% NP-40 and 25 mM DTT) followed by the addition of 100 μl 4 × SDS loading buffer. For signaling analysis, the BMDM supernatants were removed at the indicated time points, and cells were washed once with PBS, after which cells were lysed with RIPA buffer. Proteins from lung tissues were extracted using RIPA buffer supplemented with protease and phosphatase inhibitors (Roche), and 30 μg per sample was loaded on the gel. Proteins were separated by electrophoresis through 8%–12% polyacrylamide gels. Following electrophoretic transfer of proteins onto PVDF membranes (Millipore, IPVH00010), non-specific binding was blocked by incubation with 5% skim milk; then membranes were incubated with the following primary antibodies: anti-caspase-1 (AdipoGen, AG-20B-0042, 1:1000), anti-caspase-3 (CST, #9662, 1:1000), anti-cleaved caspase-3 (CST, #9661, 1:1000), anti-caspase-7 (CST, #9492, 1:1000), anti-cleaved caspase-7 (CST, #9491, 1:1000), anti-caspase-8 (CST, #4927, 1:1000), anti-cleaved caspase-8 (CST, #8592, 1:1000), anti-pRIPK3 (CST, #91702S, 1:1000), anti-RIPK3 (ProSci, #2283, 1:1000), anti-pMLKL (CST, #37333, 1:1000), anti-MLKL (Abgent, AP14272b, 1:1000), anti-GSDMD (Abcam, ab209845, 1:1000), anti-GSDME (Abcam, ab215191, 1:1000), anti-pSTAT1 (CST, #7649, 1:1000), anti-STAT1 (CST, #14994, 1:1000), anti-ZBP1 (AdipoGen, AG-20B-0010, 1:1000), anti-β-actin (Proteintech, 66009-1-IG, 1:5000), human anti-caspase-1 (R&D systems; Cat# MAB6215, 1:1000), human anti-caspase-8 (Enzo, ALX-804-242, 1:1000), human anti-GSDMD (Abcam, ab210070, 1:1000), human anti-tMLKL (Abcam, ab184718, 1:1000), human anti-β-actin (CST, 4970, 1:1000), human anti-ZBP1 (R&D systems; Cat# AF6309, 1:1000) and anti-ISG15 (Santa Cruz, sc-166755). Membranes were then washed and incubated with the appropriate horseradish peroxidase (HRP)–conjugated secondary antibodies (1:5000 dilution; Jackson Immuno Research Laboratories, anti-rabbit [111-035-047], anti-mouse [315-035-047]) for 1 hour. Proteins were visualized by using Luminata Forte Western HRP Substrate (Millipore, WBLUF0500), and membranes were developed with an Amersham imager.

### Real-time cell death analysis

Real-time cell death assays were performed using an IncuCyte S3 or IncuCyte SX5 imaging system (Sartorius). BMDMs were seeded in 12-well plates (10^6^ cells/well) and stimulated. After infection, 100 nM Sytox Green (Thermo Fisher Scientific, S7020) was added. The images were acquired every 1 hour at 37°C and 5% CO_2_. The resulting images were analyzed using the software package supplied with the IncuCyte imager, which counts the number of Sytox Green-positive BMDM nuclei (Sytox^+^ BMDM nuclei) present in each image.

### Cytokine analysis

Cytokines were detected by using multiplex ELISA (Millipore, MCYTOMAG-70K) or IL-18 ELISA (Invitrogen, BMS618-3) according to the manufacturer’s instructions.

### Microarray and RNAseq analysis

For analysis of gene expression in patients with COVID-19, publicly available datasets were downloaded from the Gene Expression Omnibus database (GSE147507 (*18*), GSE171430 (*95*), GSE157103 (*96*) and GSE171110 (*97*)). The DESeq2 v1.32.0 (*98*) package was used to read the count matrices and remove genes which were not expressed in at least one sample, and the limma v3.48.3 package (*98*, *99*) in R v4.1.1 was used to determine differential expression. The Benjamini-Hochberg (*100*) adjusted *P* value < 0.05 and |log_2_FC| > 1 were then used to identify the differentially expressed genes. Genes that were differentially expressed in each of the 4 datasets were then used to form a consensus set of 31 genes consistently expressed differentially and with significance across the 4 COVID-19–related datasets. This gene set was used for subsequent analyses.

Unnormalized RNAseq data was obtained from BioProject (PRJNA638753 (*34*)) to compare non-critically ill (NCI) with critically ill (CI) patients with COVID-19. Quality control steps were performed, including normalizing quantiles using the ‘normalize.quantiles’ function from preprocessCore v1.54.0 package, followed by log_2_ transformation for downstream pathway enrichment analysis. Hallmark pathways were downloaded as genesets from MSigDB (*35*); MSigDB defines these as follows: hallmark gene sets summarize and represent specific well-defined biological states or processes and display coherent expression and were generated by a computational methodology based on identifying overlaps between gene sets in other MSigDB collections and retaining genes that display coordinate expression (*36*). Gene Set Enrichment Analysis (GSEA) (*101*) was performed using the fgsea v1.18.0 package (*102*) in R to determine the normalized enrichment scores (NES) for each pathway along with the significance of the enrichment. The average expression profile was also determined as a Z-score for the 31 genes identified above, and a heatmap was generated using the ComplexHeatmap v2.8.0 package (*103*).

For analysis of expression in Calu-3 cells infected with SARS-CoV-2 over time, the log_2_FC information at genome-wide scale was downloaded from GEO (GSE157490 (*41*)), and the mRNA data for Calu-3 cells infected with IAV over time were also downloaded from GEO (GSE76599 (*104*)). Quantile normalization and log_2_ transformation was performed on the IAV mRNA data to obtain the log_2_FC for each gene by comparing the infection vs mock sample expression profiles. The ‘fgsea’ function was then used to estimate the NES for IFN signaling pathways (IFN-ɑ and IFN-γ responses) at each available time-point for the SARS-CoV-2 and IAV infection, respectively.

For comparison of gene expression in the blood transcriptome between patients with SARS-CoV-2 infection and influenza and sepsis, we downloaded RNAseq data from GEO (GSE163151 (*39*)). Quality control steps were performed, including normalizing quantiles using the ‘normalize.quantile’ function from preprocessCore followed by log_2_ transformation. Comparisons were made for COVID-19 patient expression profiles vs healthy controls, sepsis vs healthy controls and Influenza vs healthy controls to determine the log_2_FC for all genes. The set of 50 aforementioned hallmark pathways and the log_2_FC information of each gene were used in the ‘fgsea’ function to obtain the NES for each pathway. The average expression profile was also determined as a Z-score for the 31 consensus genes identified above, and a heatmap was generated.

### Generation of Brie lentiviral library

The Mouse Brie CRISPR KO library was a gift from David Root and John Doench (Addgene #73632 and #73633). The plasmid library was amplified and validated in the Center for Advanced Genome Engineering at St. Jude as described in the Broad GPP protocol, the only exception being the use of Endura DUOs electrocompetent cells. The St. Jude Hartwell Center Genome Sequencing Facility provided all NGS sequencing. Single end 100 cycle sequencing was performed on a NovaSeq 6000 (Illumina). Validation to check gRNA presence and representation was performed using calc_auc_v1.1.py (https://github.com/mhegde/) and count_spacers.py (*105*). Viral particles were produced by the St. Jude Vector Development and Production laboratory. CRISPR KO screens were analyzed using Mageck-Vispr/0.5.7 (*106*).

### CRISPR/CAS9 library screen

CAS9-expressing immortalized BMDMs (iBMDMs) generated from *Cas9-GFP* knock-in mice (*107*) were infected with the lentiviral particles at an MOI of 0.3. Two replicates of an adequate number of cells were used as control to obtain a representation (screen depth) of > 500 cells for each sgRNA of the library, and a similar number of cells from the same batch of virus preparation were infected with MHV at a multiplicity of infection (MOI) of 0.2 for 24 hours. The uninfected control cell population and the surviving cells from the MHV-infected samples were subjected to CRISPR screen enrichment analysis. Total genomic DNA was isolated using NucleoSpin® Blood kits (Takara Bio Inc., USA; 740954 and 740950) and the concentrations of the isolated gDNA samples were measured using NanoDrop (Thermo Fisher Scientific, USA).

Using the MAGeCK pipeline (*108*), we robustly estimated the log_2_FC with significance of the essential genes from our CRISPR screen. The genes with positive fold change were imperative for cell death. The top gene hits along with their significance from the CRISPR screen were highlighted using a volcano plot.

### Single cell analysis

Single cell data for comparisons of patients with COVID-19 vs healthy patients were obtained from FigShare (https://doi.org/10.6084/m9.figshare.14938755) (*109*). The dataset consisted of 268,745 cells; 114,201 cells from adults, and 154,544 cells from children. Only data from adults were included in the final analysis. Additional metadata information was also obtained, including information about cell types and the 2D-coordinates of the Unified Manifold Approximation and Projection (UMAP) plot. ‘MC_Basophil’, ‘Neutrophil’, ‘mDC’, ‘CD11c_mDC’, ‘pDC’, ‘moMa’, ‘nrMa’, ‘rMa’, ‘NK’, ‘IL17A_CD4’, ‘IL17A_CD8’, ‘CD8_Tm’, ‘Treg’, ‘B_cell’ and ‘Plasma_cell’ as defined in (*109*) were selected for further analyses, reducing the total number of cells to 19,361. Relabeling was performed: ‘MC_Basophil’ → ‘Basophils’, [‘mDC’, ‘CD11c_mDC’, ‘pDC’] → ‘Dendritic cells’’, [‘moMa’, ‘nrMa’, ‘rMa’] → ‘Macrophages’, ‘NK’ → ‘NK cells’, ‘IL17A_CD4’ → ‘CD4 T cells’, [‘IL17A_CD8’, ‘CD8_Tm’] → ‘CD8 T cells’, ‘B_cell’ → ‘B cells’ and ‘Plasma_cell’ → ‘Plasma cells’, respectively. The revised dataset contained 16,013 cells from COVID-19 patients and 3,348 healthy cells from adults. A nonparametric Mann-Whitney-Wilcoxon test (*110*) was used to compare average *ZBP1* expression across COVID-19 vs healthy cells.

Single cell data for comparisons of patients with stable vs progressive COVID-19 were obtained from GEO (GSE155224 (*67*)). The dataset consisted of 42,293 cells loaded using the Seurat v4.0.2 package (*111*). The SignacX v2.2.4 package (*112*) with default settings was used to annotate the cells as ‘B.memory’, ‘B.naive’, ‘DC’, ‘Mon.Classical’, ‘Neutrophils’, ‘NK’, ‘Plasma.cells’, ‘T.CD4.memory’, ‘T.CD4.naive’, ‘T.CD8.cm’, ‘T.CD8.em’, ‘T.CD8.naive’, ‘T.regs’ and ‘Unclassified’. Cell types having < 500 cells were removed, including ‘Plasma.cells’, ‘T.regs’ and ‘T.CD8.cm’ cells; those labeled as ‘Unclassified’ cells were also removed. In total, 25,173 cells corresponding to 6 Stable COVID-19 patients and 13,248 cells corresponding to 4 Progressive COVID-19 patients were included. Relabeling was performed: ‘B.memory’ → ‘B Memory’, ‘B.naive’ → ‘B Naive’, ‘DC’ → ‘Dendritic cells’, ‘NK’ → ‘NK cells’, T.CD4.memory’ → ‘T CD4-Memory’, ‘T.CD4.naive’ → ‘T CD4-, Naive’, ‘T.CD8.cm’ → ‘T CD8-CM’, ‘T.CD8.em’ → ‘T CD8-EM’ and ‘T.CD8.naive’ → ‘T CD8-Naive’ cells respectively. We then determined the average ZBP1 expression across Stable vs Progressive COVID-19 patients bifurcated by the various cell states of interest.

### Statistical analysis

GraphPad Prism 8.0 software was used for data analysis. Data are presented as mean ± SEM. Statistical significance was determined by *t* tests (two-tailed) or Mann-Whitney-Wilcoxon test (non-parametric) for two groups or one-way or two-way ANOVA with multiple comparisons test for more groups. Survival analysis was performed using the log-rank (Mantel-Cox) test. *P* values less than 0.05 were considered statistically significant where *P < 0.05, **P < 0.01, ***P < 0.001 and ****P < 0.0001.

## Supplementary Materials

### Other Supplementary Material for this manuscript includes the following:

MDAR Reproducibility Checklist
