# Supplementary material for: ZBP1-dependent inflammatory cell death, PANoptosis, and cytokine storm disrupt IFN therapeutic efficacy during coronavirus infection
Source: Sci Immunol. 2022 May 19:eabo6294. doi: 10.1126/sciimmunol.abo6294 (PMC9161373; doi:10.1126/sciimmunol.abo6294)
Supplement: Supplementary file 1 — Figs. S1 to S8 [file sciimmunol.abo6294_sm.pdf]

Science Immunology
AAAS

Supplementary Materials for

ZBP1-dependent inflammatory cell death, PANoptosis, and cytokine storm disrupt IFN therapeutic efficacy during coronavirus infection

Rajendra Karki *et al.*

Corresponding author: Thirumala-Devi Kanneganti, Thirumala-Devi.Kanneganti@StJude.org

DOI: 10.1126/sciimmunol.abo6294

The PDF file includes:

Figs. S1 to S8

Other Supplementary Material for this manuscript includes the following:

MDAR Reproducibility Checklist
Tables S1 and S2

Supplementary figures

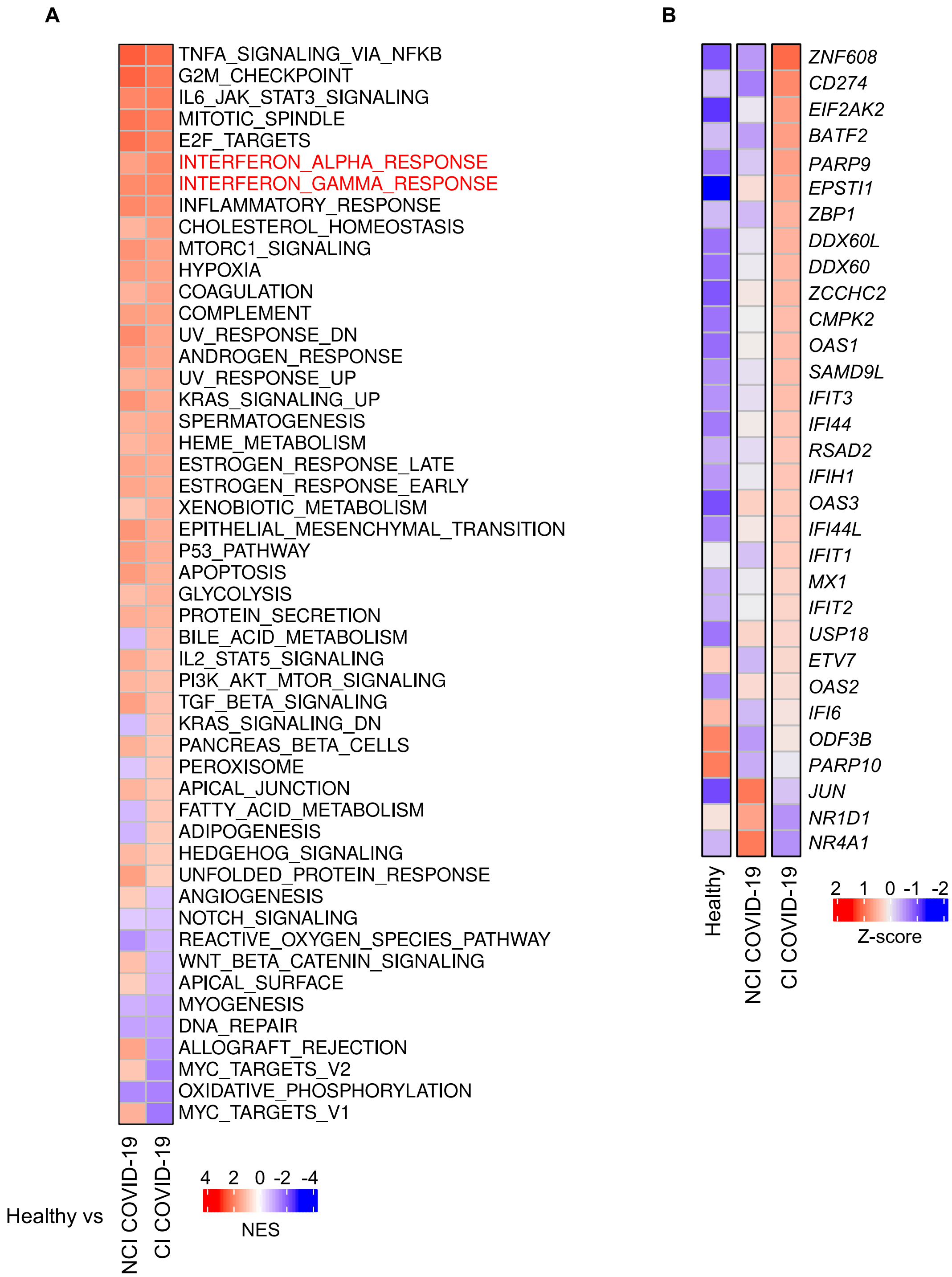

Fig. S1. Altered expression of signaling pathways and IFN responsive genes found in patients with COVID-19
(A) Heatmap depicting the altered expression of pathways in whole blood cell (WBC) transcriptomes of non-critically ill (NCI) and critically ill (CI) patients with COVID-19 compared with healthy patients (*34*). NES, normalized enrichment scores. (**B)** Heatmap depicting the differentially regulated IFN-responsive genes in WBC transcriptomes of NCI and CI patients with COVID-19 compared with healthy patients (*34*).

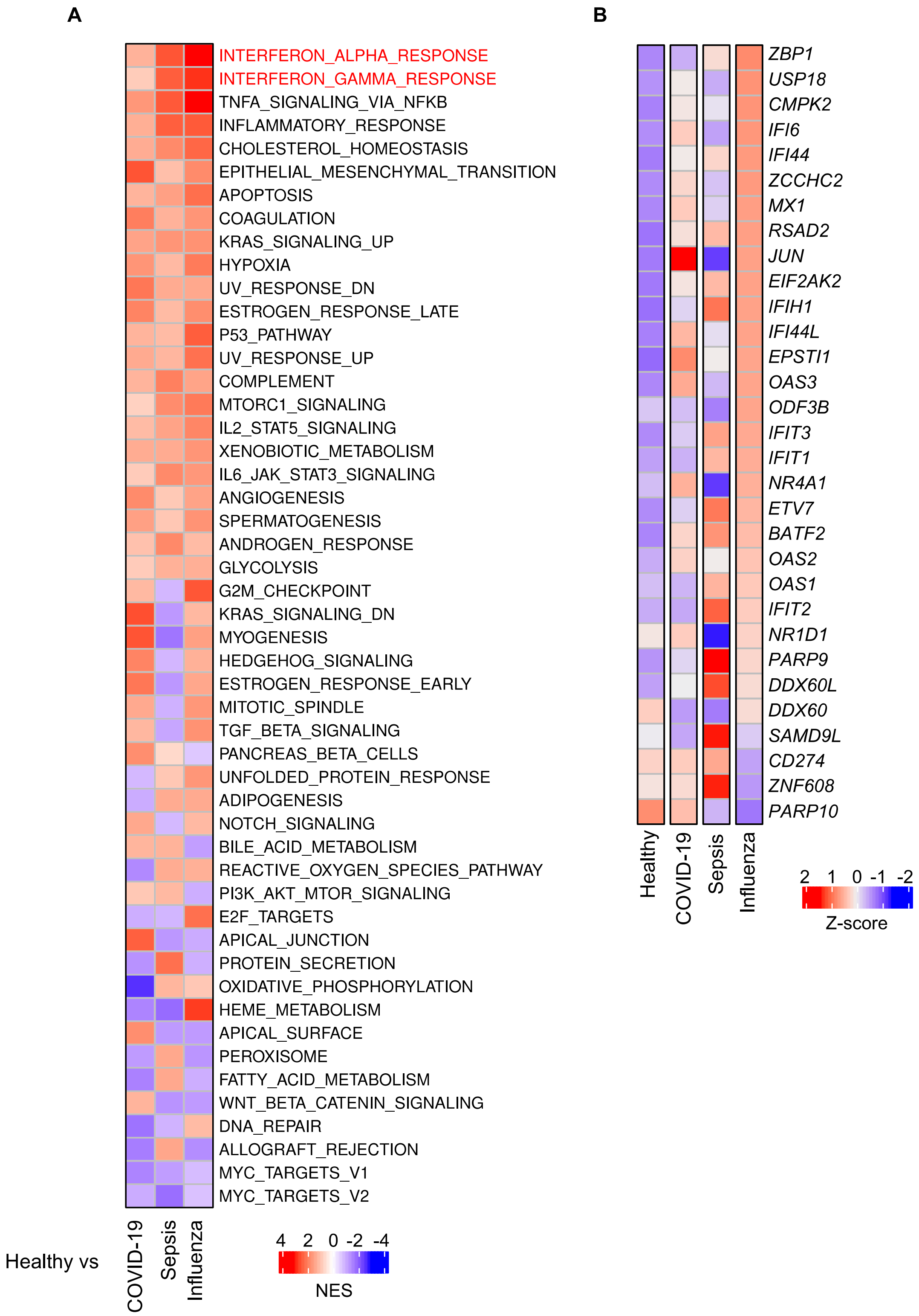

Fig. S2. Reduced IFN responses occur in patients with COVID-19

(A) Heatmap depicting the altered pathways in whole blood of patients with COVID-19, sepsis and influenza compared with that of healthy patients (*38*). NES, normalized enrichment scores. (**B**) Heatmap depicting the differentially regulated IFN-responsive genes in whole blood of patients with COVID-19, sepsis and influenza compared with that of healthy patients (*38*).

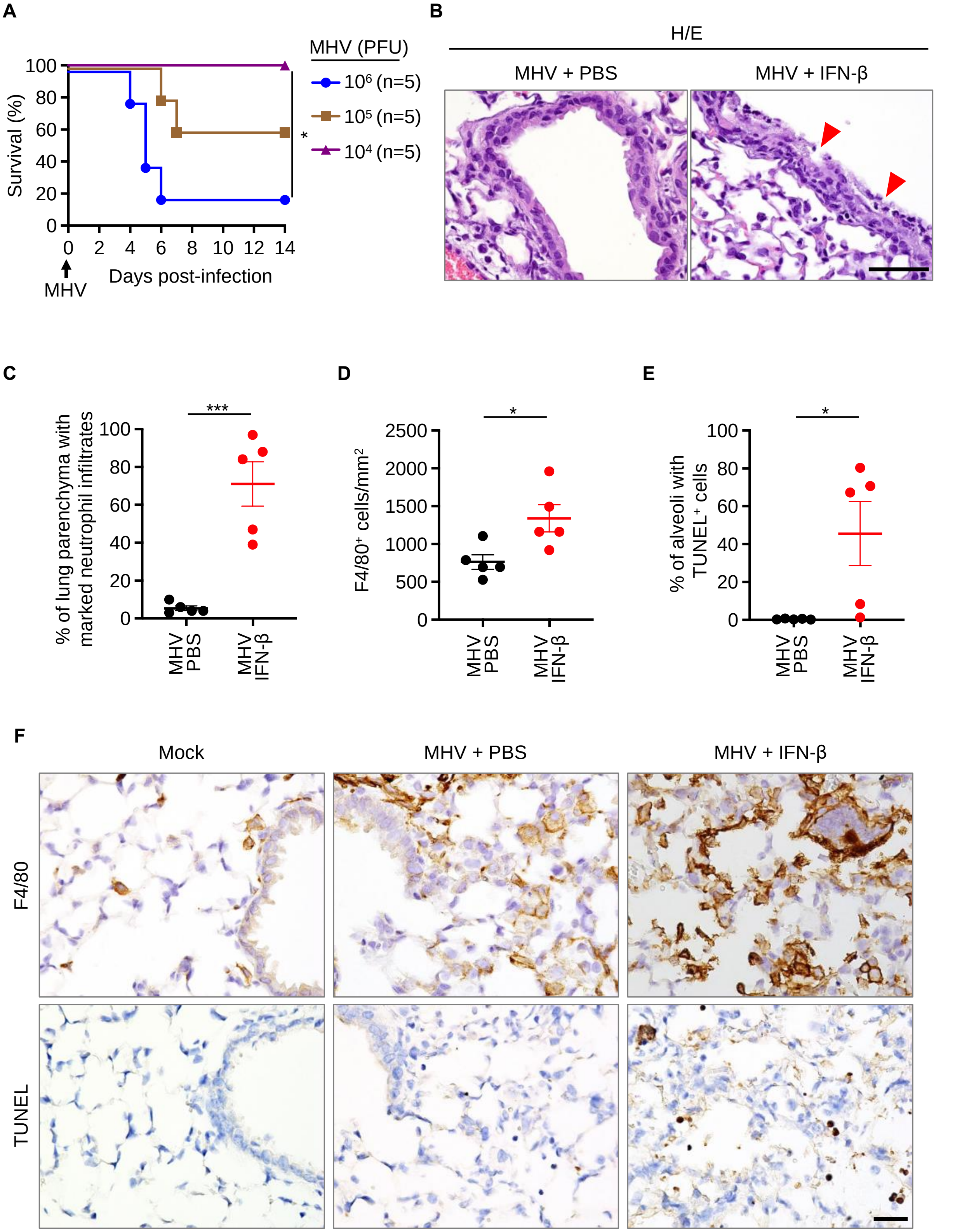

Fig. S3. IFN-β treatment induces lung pathology during β-coronavirus infection
(**A**) Survival of 6- to 8-week-old wild type (WT) mice after intranasal infection with mouse hepatitis virus (MHV) at the indicated PFUs (n = 5 for each group). **(B)** Hematoxylin and eosin (H/E) staining

of lung samples from PBS- or IFN-β–treated WT mice 3 days after MHV infection. Arrows indicate loss of bronchiolar epithelium. **(C)** Quantification of neutrophil infiltrates in lung samples from PBS- or IFN-β–treated WT mice 3 days after MHV infection based on neutrophil staining. **(D)** Quantification of F4/80-positive cells from **(F)**. **(E)** Quantification of TUNEL positive cells from **(F)**. **(F)** Staining for F4/80 and TUNEL in lung samples from mock infected and PBS- or IFN-β–treated WT mice 3 days after MHV infection. Scale bar, 50 μm. Images are representative of an experiment containing at least 5 biologically independent samples in each group. $^{*}P < 0.05$ and $^{***}P < 0.001$. Analysis was performed using the *t* test (two tailed) (C, D and E) or log-rank test (Mantel-Cox) (**A**). Each symbol represents one mouse. Data are shown as mean ± SEM.

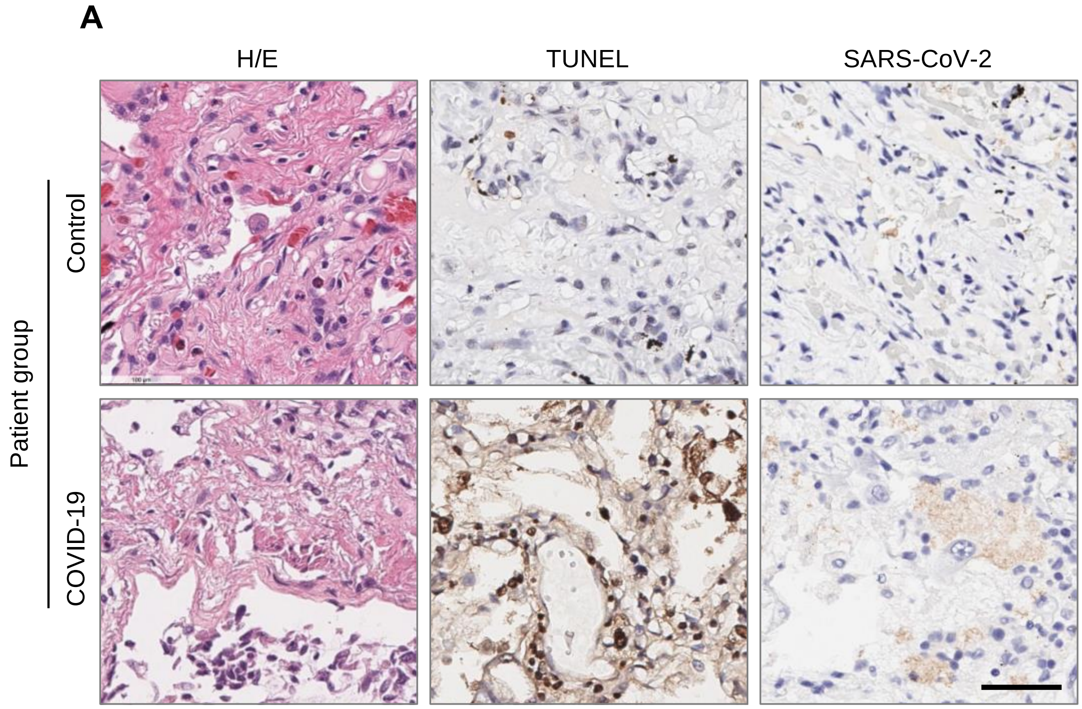

Fig. S4. Cell death and inflammation occur in the lungs of patients who succumb to COVID-19
(A) Staining for hematoxylin and eosin (H/E), TUNEL and SARS-CoV-2 nucleocapsid protein in lung samples from deceased patients without COVID-19 (Control) or with COVID-19. Scale bar, 100 µm.

A

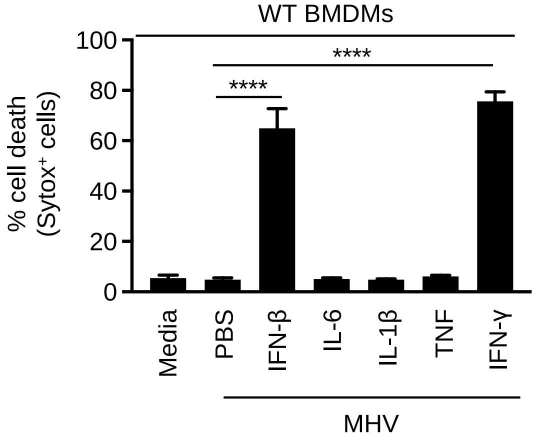

B

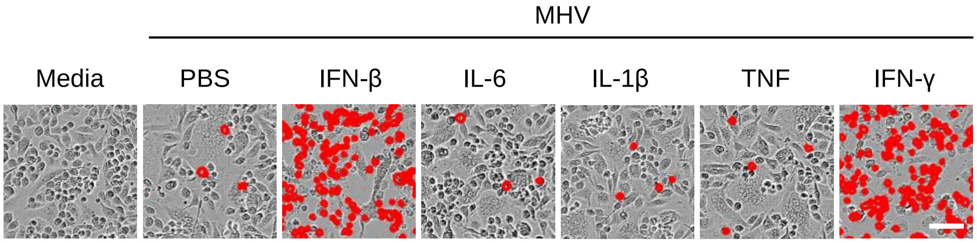

Fig. S5. Treatment with IFNs, but not other cytokines, induces robust cell death during MHV infection

(A) Cell death in PBS-, IFN-β–, IL-6–, IL-1β–, TNF- or IFN-γ–treated wild type (WT) bone marrow-derived macrophages (BMDMs) 24 hours after mouse hepatitis virus (MHV) infection. **(B)** Representative images of cell death in PBS-, IFN-β–, IL-6–, IL-1β–, TNF- or IFN-γ–treated BMDMs are shown at 24 h after MHV infection. Scale bar, 50 µm. Data are representative of at least three independent experiments. ****$P < 0.0001$. Analysis was performed using the one-way ANOVA **(A)**. Data are shown as mean ± SEM.

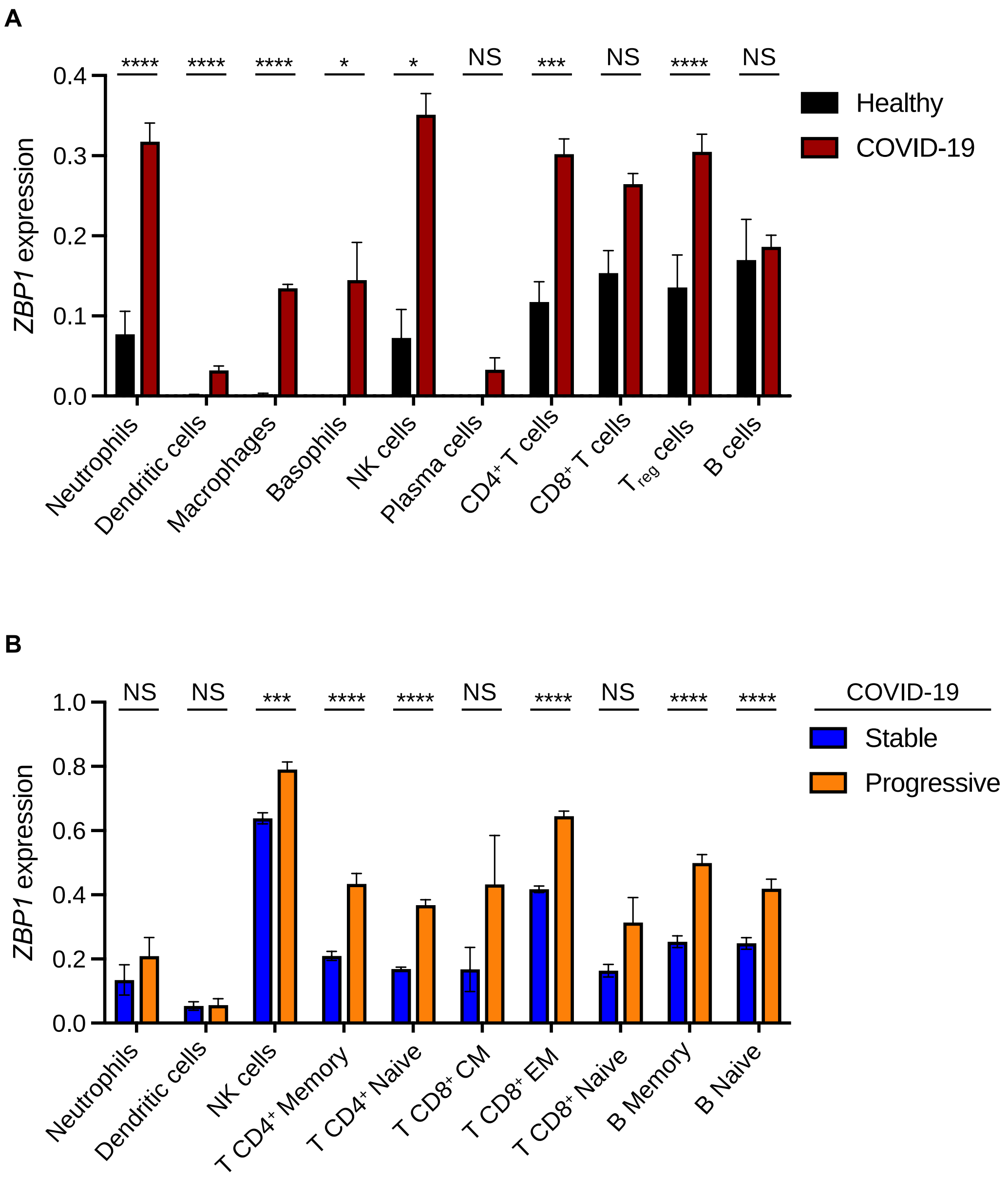

Fig. S6. *ZBP1* expression is increased in immune cells of patients with COVID-19, especially patients with progressive disease

(**A**) *ZBP1* expression in neutrophils, dendritic cells, macrophages, basophils, natural killer (NK0 cells, plasma cells, CD4$^+$ T cells, CD8$^+$ T cells, T_{reg} cells and B cells from patients with COVID-19 compared with healthy controls. (**B**) *ZBP1* expression in neutrophils, dendritic cells, NK cells, T CD4$^+$ memory, T CD4$^+$ naïve, T CD8$^+$ CM, T CD8$^+$ EM, T CD8$^+$ naïve, B memory and B naïve cells from patients with stable or progressive COVID-19 (*66*). NS, no significance; *$P < 0.05$, ***$P < 0.001$ and ****$P < 0.0001$. Analysis was performed using the nonparametric Mann-Whitney-Wilcoxon test. Data are shown as mean ± SEM.

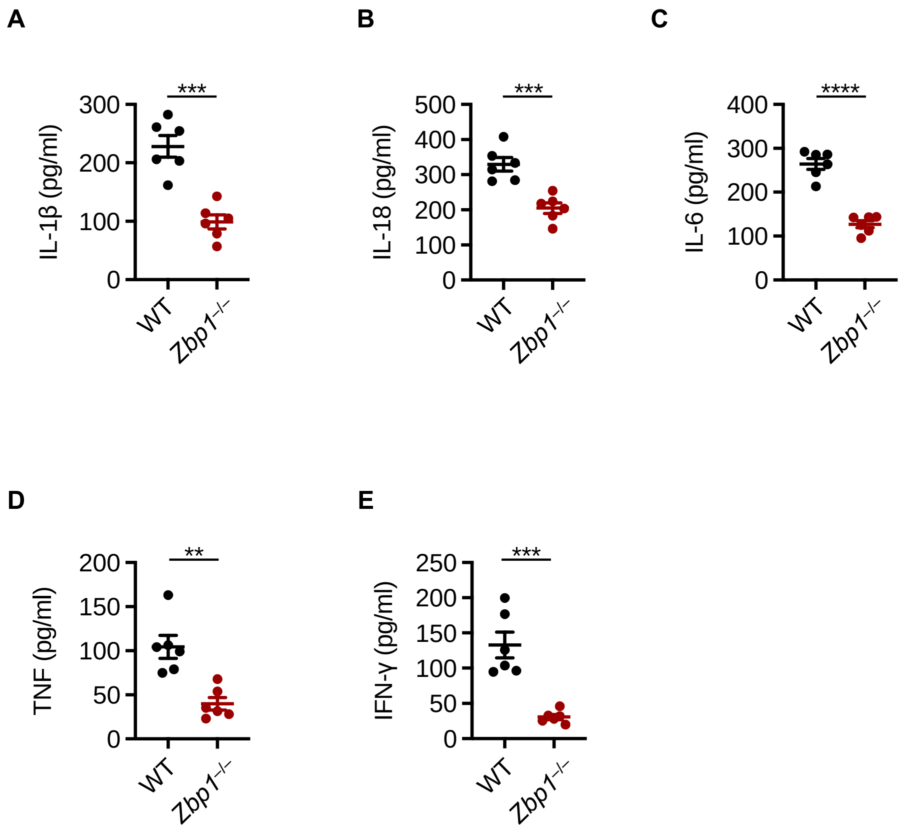

Fig. S7. IFN-β–driven cytokine production depends on ZBP1 during β-coronavirus infection

Analysis of (**A**) IL-1β, (**B**) IL-18, (**C**) IL-6, (**D**) TNF and (**E**) IFN-γ levels in BALF of IFN-β–treated wild type (WT) (n = 6) or *Zbp1*$^{-/-}$ mice (n = 6) 3 days after mouse hepatitis virus (MHV) infection. Data are representative of at least two independent experiments. $^{**}P < 0.01$, $^{***}P < 0.001$ and $^{****}P < 0.0001$. Analysis was performed using the *t* test (two tailed). Each symbol represents one mouse. Data are shown as mean ± SEM.

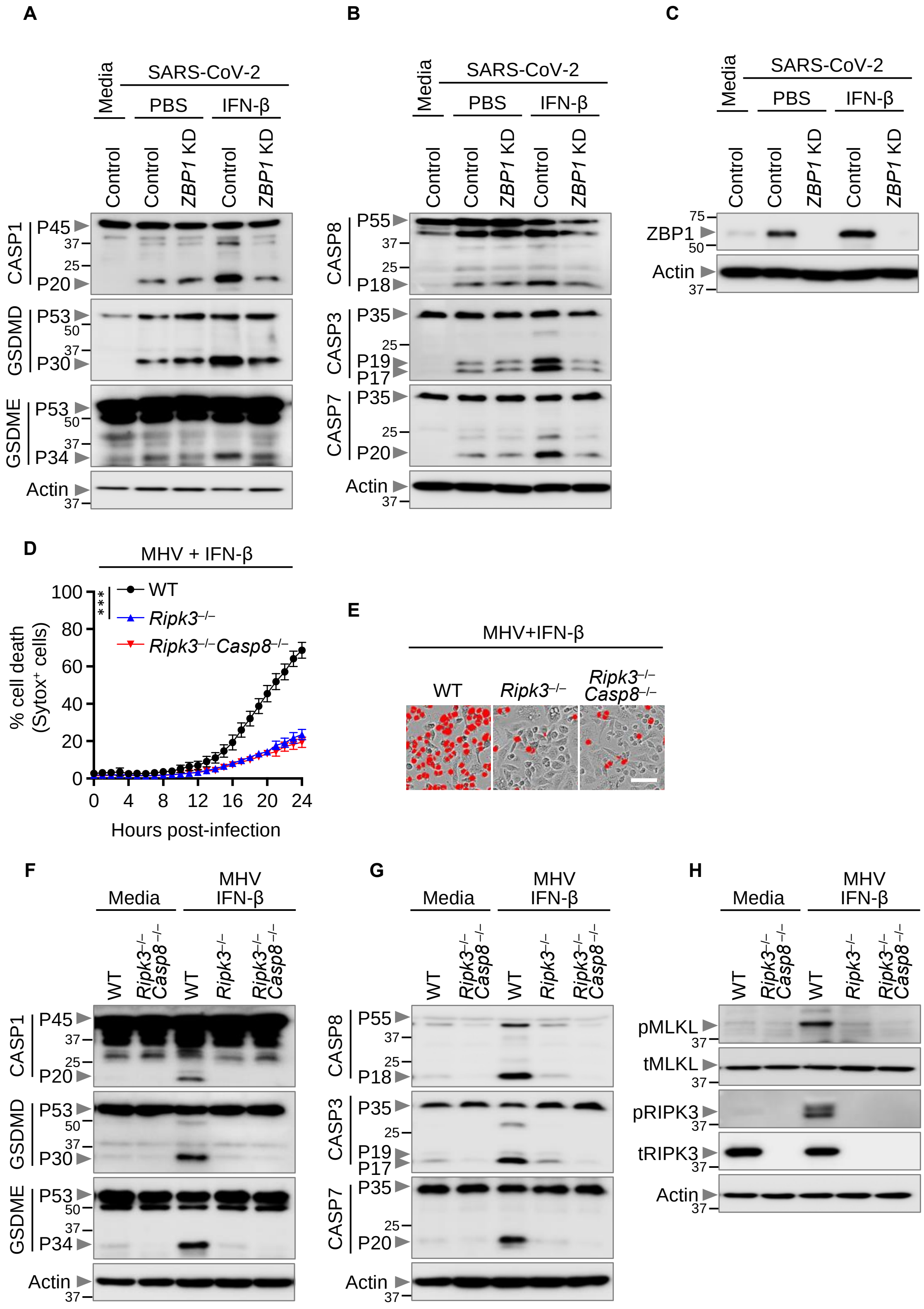

Fig. S8. IFN-β–driven cell death, PANoptosis, depends on the ZBP1-RIPK3 axis during β-coronavirus infection

(**A–C**) Immunoblot analysis of (**A**) pro- (P45) and activated (P20) caspase-1 (CASP1); pro- (P53) and activated (P30) gasdermin D (GSDMD), pro- (P53) and activated (P34) gasdermin E

(GSDME); (**B**) pro- (P55) and cleaved caspase-8 (CASP8; P18), pro- (P35) and cleaved caspase-3 (CASP3; P19 and P17) and pro- (P35) and cleaved caspase-7 (CASP7; P20); and (**C**) ZBP1 in THP-1 (Control) and *ZBP1*-silenced (*ZBP1* KD) THP-1 cells treated with PBS or IFN-β 48 h after SARS-CoV-2 infection. (**D**) Real-time analysis of cell death in mouse hepatitis virus (MHV)-infected wild type (WT), *Ripk3*$^{–/–}$ and *Ripk3*$^{–/–}$*Casp8*$^{–/–}$ bone marrow-derived macrophages (BMDMs) in the presence of IFN-β. (**E**) Representative images of cell death in IFN-β–treated WT, *Ripk3*$^{–/–}$ and *Ripk3*$^{–/–}$*Casp8*$^{–/–}$ BMDMs are shown at 24 h after MHV infection. Scale bar, 50 μm. (**F–H**) Immunoblot analysis of (**F**) pro- (P45) and activated (P20) CASP1, pro- (P53) and activated (P30) GSDMD, pro- (P53) and activated (P34) gasdermin E (GSDME); (**G**) pro- (P55) and cleaved CASP8 (P18), pro- (P35) and cleaved CASP3 (P19 and P17) and pro- (P35) and cleaved CASP7 (P20); and (**H**) phosphorylated MLKL (pMLKL), total MLKL (tMLKL), phosphorylated RIPK3 (pRIPK3) and total RIPK3 (tRIPK3) in IFN-β–treated WT, *Ripk3*$^{–/–}$ and *Ripk3*$^{–/–}$*Casp8*$^{–/–}$ BMDMs 24 h after MHV infection. Molecular weight marker sizes in kDa are indicated in small font on the left of each blot. Actin was used as the internal control. Data are representative of at least three independent experiments. ***$P < 0.001$. Analysis was performed using the two-way ANOVA (**D**). Data are shown as mean ± SEM.
